# DISC1 regulates N-methyl-D-aspartate receptor dynamics: abnormalities induced by a *Disc1* mutation modelling a translocation linked to major mental illness

**DOI:** 10.1038/s41398-018-0228-1

**Published:** 2018-09-06

**Authors:** Elise L. V. Malavasi, Kyriakos D. Economides, Ellen Grünewald, Paraskevi Makedonopoulou, Philippe Gautier, Shaun Mackie, Laura C. Murphy, Hannah Murdoch, Darragh Crummie, Fumiaki Ogawa, Daniel L. McCartney, Shane T. O’Sullivan, Karen Burr, Helen S. Torrance, Jonathan Phillips, Marion Bonneau, Susan M. Anderson, Paul Perry, Matthew Pearson, Costas Constantinides, Hazel Davidson-Smith, Mostafa Kabiri, Barbara Duff, Mandy Johnstone, H. Greg Polites, Stephen M. Lawrie, Douglas H. Blackwood, Colin A. Semple, Kathryn L. Evans, Michel Didier, Siddharthan Chandran, Andrew M. McIntosh, David J. Price, Miles D. Houslay, David J. Porteous, J. Kirsty Millar

**Affiliations:** 1 Centre for Genomic and Experimental Medicine, MRC Institute of Genetics and Molecular Medicine at the University of Edinburgh, Edinburgh, UK; 2 Xtuit Pharmaceuticals, Waltham, MA USA; 3 MRC Human Genetics Unit, MRC Institute of Genetics and Molecular Medicine at the University of Edinburgh, Edinburgh, UK; 4 Molecular Pharmacology Group, Wolfson Building, Institute of Neuroscience and Psychology, The University of Glasgow, University Avenue, Glasgow, UK; 5 Centre for Regenerative Medicine, The University of Edinburgh, Edinburgh, UK; 6 Translational In Vivo Models at Sanofi, Frankfurt, Germany; 7 Division of Psychiatry, The University of Edinburgh, Edinburgh, UK; 8 Regeneron Pharmaceuticals, Tarrytown NY, USA; 9 Translational Sciences at Sanofi, Chilly-Mazarin, France; 10 Centre for Integrative Physiology, The University of Edinburgh, Edinburgh, UK; 11 School of Cancer and Pharmaceutical Sciences, King’s College London, London, UK

## Abstract

The neuromodulatory gene *DISC1* is disrupted by a t(1;11) translocation that is highly penetrant for schizophrenia and affective disorders, but how this translocation affects DISC1 function is incompletely understood. N-methyl-D-aspartate receptors (NMDAR) play a central role in synaptic plasticity and cognition, and are implicated in the pathophysiology of schizophrenia through genetic and functional studies. We show that the NMDAR subunit GluN2B complexes with DISC1-associated trafficking factor TRAK1, while DISC1 interacts with the GluN1 subunit and regulates dendritic NMDAR motility in cultured mouse neurons. Moreover, in the first mutant mouse that models DISC1 disruption by the translocation, the pool of NMDAR transport vesicles and surface/synaptic NMDAR expression are increased. Since NMDAR cell surface/synaptic expression is tightly regulated to ensure correct function, these changes in the mutant mouse are likely to affect NMDAR signalling and synaptic plasticity. Consistent with these observations, RNASeq analysis of the translocation carrier-derived human neurons indicates abnormalities of excitatory synapses and vesicle dynamics. RNASeq analysis of the human neurons also identifies many differentially expressed genes previously highlighted as putative schizophrenia and/or depression risk factors through large-scale genome-wide association and copy number variant studies, indicating that the translocation triggers common disease pathways that are shared with unrelated psychiatric patients. Altogether, our findings suggest that translocation-induced disease mechanisms are likely to be relevant to mental illness in general, and that such disease mechanisms include altered NMDAR dynamics and excitatory synapse function. This could contribute to the cognitive disorders displayed by translocation carriers.

## Introduction

N-methyl-D-aspartate receptors (NMDAR) are vital for synaptic plasticity and cognitive processes, and are strongly implicated in the pathophysiology of schizophrenia in particular^1^. *GRIN2A*, encoding the NMDAR GluN2A subunit, was recently reported to show genome-wide significant association with schizophrenia^2^, while genomic copy number variants (CNVs) enriched in schizophrenia patients target *GRIN1*, encoding the GluN1 subunit^3,4^. Such findings support a role for NMDAR in psychiatric disorders, but direct mechanistic insight is still required.

NMDAR function is regulated at many stages, including subunit expression and composition, and dynamic modulation of surface and synaptic levels. The latter can be elicited through control of NMDAR forward trafficking to the plasma membrane, subsequent insertion into synapses, or endocytosis^5^. The obligatory GluN1 subunit is incorporated into all NMDAR, along with other subunit types including GluN2A/B. GluN1 is synthesised in excess and stored within the endoplasmic reticulum (ER), where NMDAR are assembled prior to transportation to the Golgi and onwards to the cell surface^5^. Every stage of NMDAR forward trafficking is tightly modulated to ensure that the required quantity of receptors is present at the cell surface and synapse^5^. Genetic dysregulation of forward trafficking would be predicted to adversely affect the NMDAR function, with knock-on effects for synapse strength and plasticity^5^.

DISC1 is disrupted in a large, multi-generation family by a chromosomal t(1;11) translocation that is linked to major mental illness^6–8^. DISC1 is critical for several processes in the developing and adult brain^9,10^, and has been connected to NMDAR function through regulation of downstream processes that underlie synaptic plasticity^11,12^. DISC1 also regulates neuronal microtubule-based cargo transport, including trafficking of mitochondria, synaptic vesicles and messenger RNAs^13–15^, and associates with the motor protein adaptors TRAK1 and TRAK2^16,17^.

Here we demonstrate direct interaction between GluN1 and DISC1, and association between GluN2B and TRAK1. Applying a novel live-imaging method, we demonstrate that DISC1 regulates dendritic NMDAR motility. Utilising a mouse model of DISC1 disruption by the translocation, we show that mutant mouse neurons exhibit increased NMDAR fast active transport and cell surface/synaptic NMDAR expression. These observations implicate dysregulated NMDAR dynamics/signalling and excitatory synapse dysfunction in the psychiatric disorders displayed by translocation carriers. In further support of a general disease mechanism revealed by the translocation, we find that it impacts the biological pathways highlighted by independent schizophrenia and depression GWAS and CNV studies.

## Materials and methods

Detailed materials and methods for all experiments are provided in the Supplementary information. Essential information is included below.

### Generation of induced pluripotent stem cells (IPSC) from dermal fibroblasts

Dermal fibroblasts were reprogrammed using non-integrating episomal plasmids incorporating Oct3/4, shRNA to p53, SOX2, KLF4, L-MYC and LIN28^18^, and converted to neural precursor cells by dual-SMAD signalling inhibition^19^.

### Generation of a mouse model of the t(1;11) translocation

Mice were genetically engineered using Regeneron’s GEMM platform (VelociMouse®). *Disc1* was targeted in embryonic stem cell clones using a vector containing mouse *Disc1* genomic DNA encompassing exons 6, 7 and 8 as the 5′ homologous arm, and sequences 3′ of *Disc1* as the 3′ homologous arm. Human chromosome 11 genomic DNA, encompassing putative exons 4, 5, 6, 7a and 7b of *DISC1FP1*^20^ followed by a loxP-flanked neo cassette, was inserted between the mouse homologous arms. Homologous recombination removed 98,550 bp of mouse *Disc1* downstream of exon 8 and inserted 114,843 bp of human *DISC1FP1* sequence. The edited endogenous mouse *Disc1* locus mimics the *DISC1*/*DISC1FP1* gene fusion event on the derived chromosome 1 in translocation carriers, and the mutation is referred to as *Der1*.

### RNA sequencing

Three control and three translocation-carrying neural precursor lines were each differentiated to neurons independently in triplicate. RNA was extracted and sequenced to a depth of 60 million paired-end reads. Differential gene expression was analysed at the whole gene and single exon levels using DESeq2 from the R statistical package^21^ and DEXSeq^22^, respectively.

### Dendra2 NMDAR trafficking assay

A Dendra2 tag was fused to the GluN1 C terminus. Dendra2 exhibits green fluorescence, but can be photoconverted to red fluorescence. DIV7 hippocampal neurons were co-transfected with GluN1–Dendra2 plus HA–GluN2B to maximise NMDAR assembly, and therefore trafficking outwith the ER. Dendra2 was photoconverted within a region of interest positioned on a primary dendrite. Time-lapse images were captured every 15 s for 3 min, and red fluorescence bidirectional movement along dendrites was quantified. Essentially, dendrites were divided into 5-μm bins, and red fluorescence intensity was quantified within each bin at each timepoint. Finally, the fluorescence intensity within each bin was normalised to dendritic area and fluorescence intensity in bin zero at time zero.

Fluorescence peak average velocities were determined individually for each neuron using the 10-μm and 15-μm bins where the peak was clearly identifiable. Maximum velocity estimates were determined individually for each neuron using the 25–40-μm bins, where the appearance of the leading edge of the wave of red fluorescence could be ascertained.

## Results

### Neural precursor cells (NPC) and neurons derived from t(1;11) translocation carriers

Dermal fibroblasts from translocation carriers diagnosed with schizophrenia, recurrent major depression, cyclothymia or recurrent major depression plus bipolar disorder (not otherwise specified) and karyotypically normal family members (Supplementary Figure 1) were reprogrammed using non-integrating episomes^18^. NPC lines were generated from the IPSC (Supplementary Figure 2a–c) as described previously^19^. All NPC lines exhibit typical morphology and form rosettes in the culture, with almost 100% of cells expressing the neural progenitor marker NES (Supplementary Figure 2c). Each NPC line consistently differentiates to produce neurons expressing the neuronal marker βIII-tubulin (Supplementary Figure 2d).

**Fig. 1:**
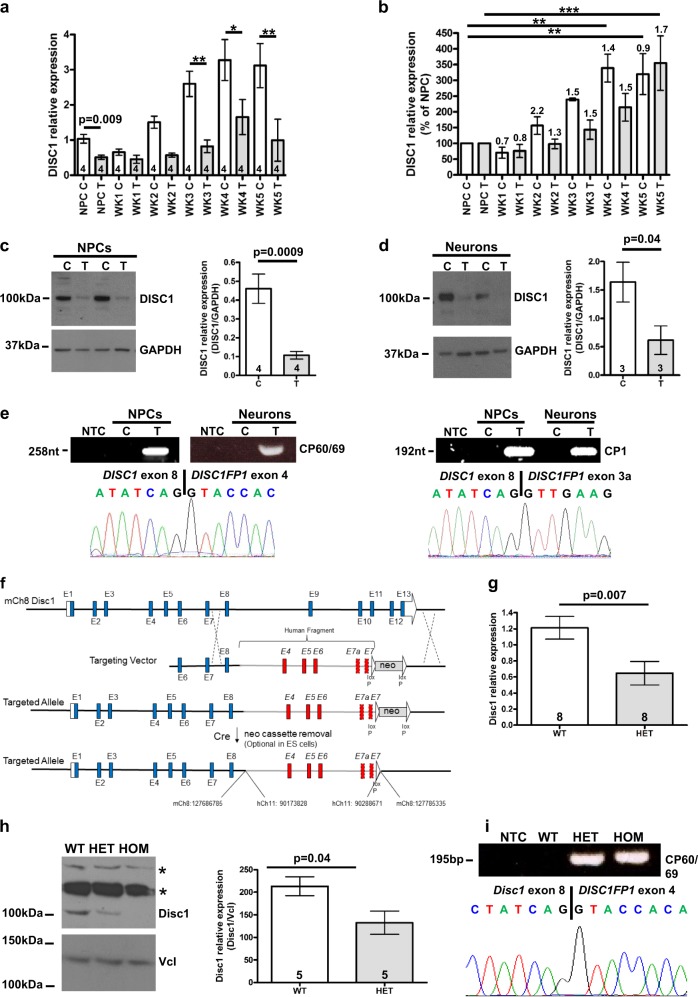
DISC1 expression in neural precursors and neurons derived from members of the t(1;11) family by the IPSC route, and in the *Der1* mouse model. **a** Quantitative RT-PCR analysis of DISC1 transcripts in NPCs and differentiating neurons over 5 weeks. Data analysed by two-tailed *t* test (NPC samples only) and one-way ANOVA (all samples, *p* < 0.0001), with pairwise Bonferroni post-test. **b** Data from a expressed as percentage of the control. Data analysed by one-way ANOVA (*p* < 0.0001) with pairwise Bonferroni post-test between NPC and each week of differentiation. Numbers above the columns indicate fold-change in comparison to the previous week. **c** and **d** Immunoblotting of DISC1 protein in neural precursors, (**c**), or NPCs differentiated to neurons in triplicate (**d**). Left, representative immunoblots; right, densitometric analysis. Data analysed by two-tailed *t* test. **e** Detection of chimeric CP60/CP69 and CP1 transcripts by RT-PCR and sequencing. **f** Schematic of the mouse endogenous *Disc1* allele targeting strategy. **g** Quantitative RT-PCR analysis of *Disc1* transcripts in adult mouse whole brain. Data analysed by two-tailed *t* test. **h** Disc1 protein expression in adult mouse whole brain. Left, representative immunoblot; right, quantification. Data analysed by two-tailed *t* test. ns, non-specific bands. **i** Detection of chimeric CP60/CP69 transcripts by RT-PCR and sequencing. NTC non-template control, C control, T translocation carrier, WT *Disc1*^*wt/wt*^; HET, *Disc1*^*wt/Der1*^; HOM and *Disc1*^*Der1/Der1*^. Error bars represent SEM; *p < 0.05; **p < 0.01 and ***p < 0.001; *n* indicated on graphs

To examine the effect of the translocation upon DISC1 transcript levels in the t(1;11) family NPCs and neurons, NPCs were differentiated over 5 weeks, with sampling at time zero (before differentiation onset) and weekly thereafter, to obtain the RNA for quantitative RT-PCR analysis. Wild-type DISC1 expression is decreased in translocation-carrying NPCs and neurons relative to controls (Fig. 1a). Moreover, DISC1 expression increases as differentiation progresses (Fig. 1a, b), although the proportional change in each week is not altered by the translocation (Fig. 1b). DISC1 expression may therefore be particularly important in neurons. An antibody that detects full-length DISC1 (Supplementary Figure 3a) confirmed its reduced expression in NPCs and neurons (Fig. 1c, d).

Chimeric CP1 and CP60/69 transcripts, resulting from translocation-induced *DISC1* gene fusion to the *DISC1FP1* (otherwise known as *Boymaw*) gene on the derived chromosome 1^20^, were PCR-amplified from NPCs and neurons derived from translocation carriers (Fig. 1e). These transcripts encode aberrant forms of DISC1, which, when exogenously expressed, are deleterious^20^, thus they are likely damaging to the brain in vivo. However, their endogenous expression awaits confirmation.

*DISC2* and *DISC1FP1*, also disrupted by the translocation, are expressed in the NPCs and neurons, but at low, non-quantifiable levels (Supplementary Figure 3b).

### Transcriptomic analysis of t(1;11) family neurons

**Fig. 2:**
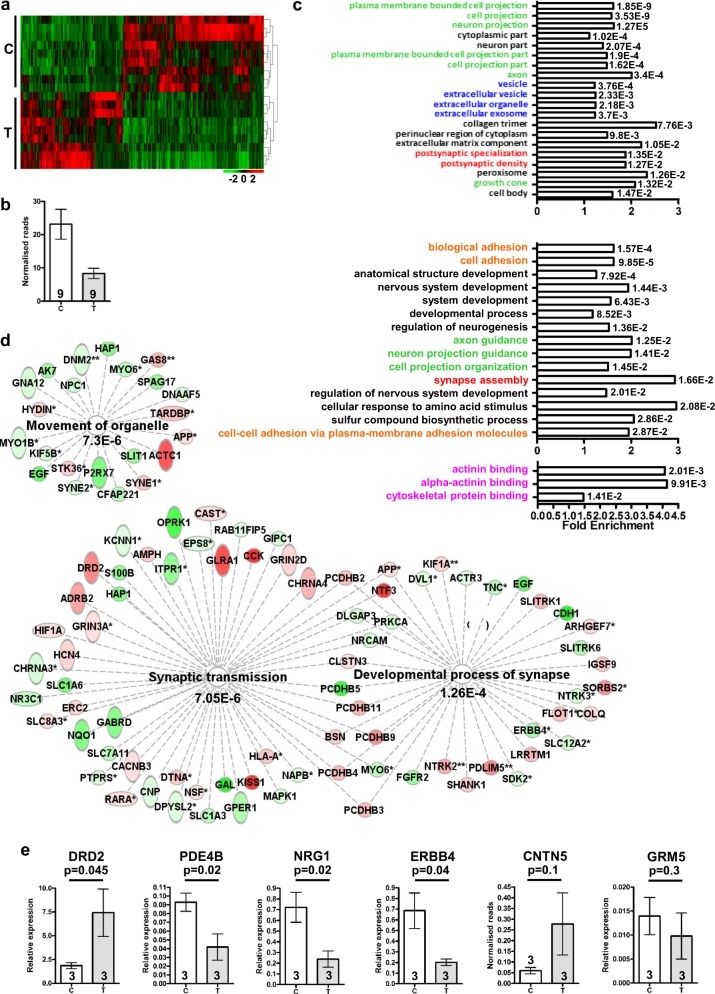
RNAseq analysis of human neurons. **a** Heatmap of differentially expressed genes (DESeq2, adjusted *p* < 0.05) in neurons from three control (28, 29, 30, Supplementary Figure 1) and three translocation lines (18, schizophrenia; 24, recurrent major depression and 55, cyclothymia, Supplementary Figure 1), three differentiations each. **b** Normalised RNASeq reads spanning DISC1 exons 8 and 9 in all samples used for RNASeq analysis. **c** Top 20 significantly enriched GO component (top), top 15 GO process (middle) plus all GO function (bottom) terms for combined DESeq2 plus DEXSeq lists, indicating FDR-corrected *q*-values. Colours indicate related GO terms. **d** Gene networks relating to synapses and organelle movement derived using DESeq2 plus DEXSeq lists in IPA. *Genes identified by DEXSeq; **genes identified by DEXSeq plus DESeq2; red, upregulated; green, downregulated. **e** Quantitative RT-PCR confirms differential expression of *DRD2*, *PDE4B*, *NRG1* and *ERBB4*, but not of *CNTN5* and *GRM5*, using three control and three translocation lines differentiated to neurons in triplicate. Data analysed by one-tailed *t* test. C control, T translocation carrier. Error bars represent SEM; *n* indicated on graphs

We next performed RNA sequencing (RNASeq) analysis as an unbiased screen for the effects of the translocation upon gene expression in the human cortical neurons. The translocation lines used were derived from patients diagnosed with schizophrenia, recurrent major depression or cyclothymia. The use of cross-diagnostic samples aimed to ensure that the major effects of the translocation are identified, rather than any disorder-specific effects due to genetic background. Overall, 1,256 (BaseMean>10) of 22,753 expressed genes were found to be differentially expressed (adjusted *p* < 0.05, Fig. 2a, Supplementary Table 1a). A total of 938 genes also exhibit differential expression of individual exons (adjusted *p* < 0.05), an indicator of altered isoform expression (Supplementary Table 1b).

IPSC-derived neuron cultures are a mix of cell types, thus it is possible that the translocation could affect gene expression patterns via influences upon cell fate. However, analysis of genes whose expressions were determined to be enriched in mouse or human astrocytes and neurons by enriched cell or single-cell RNASeq analysis^20,21^ found that only 7/41 mouse and 3/21 human astrocyte markers, and 8/25 mouse and 5/34 human neuron markers are dysregulated (Supplementary Table 1c, d). Four of the dysregulated mouse neuron markers, DLX1, DLX2, DLX5 and DLX6, are already known to be regulated by DISC1, indicating a subtle shift in neuronal properties^22,23^. Classic markers of neurons, and of inhibitory GABAergic neurons, are also unaffected (Supplementary Table 1d). Moreover, analysis of genes whose expressions are enriched in specific ‘communities’ of human excitatory and inhibitory neurons^21^ found little indication of change to either type overall (Supplementary Table 1e). Thus, the translocation does not affect the proportion of astrocytes versus neurons, or of excitatory versus inhibitory neurons, although neuron properties may be subtly altered. This is consistent with a previous study which examined the effects of a DISC1 mutation upon gene expression in IPSC-derived neurons^22^.

A total of 300 and 71 of the dysregulated genes, respectively, are also differentially expressed in IPSC-derived neurons carrying a frameshift mutation in *DISC1* exon 12^23^ (hypergeometric probability *p* = 2.5E-21 Supplementary Table 1a, b) or exon 8^22^ (*p* = 0.00002), indicating that independent *DISC1* mutations have overlapping effects. Differential *DISC1* expression was not initially detected in the whole-gene analysis, presumably due to the presence of chimeric *DISC1* transcripts originating from both derived chromosomes (Fig. 1e, Supplementary Figure 3c). However, comparison of reads that span intron 8, the location of the translocation breakpoint and that can therefore only arise from wild-type transcripts, indicates that wild-type *DISC1* transcripts are indeed reduced (Fig. 2b).

Due to the potential for positional, or other, effects of the translocation upon neighbouring gene expression, we examined the transcript levels over a region of approximately 20 Mb proximal and distal to the translocation breakpoints on chromosomes 1 and 11. Such analysis identified a number of differentially expressed genes and exons, and possible clustering of dysregulated genes around the chromosome 11 breakpoint (Supplementary Table 1f, g).

Gene ontology (GO) analysis (Fig. 2c) highlighted cell/neuron projections (seven of the top 20 GO Component terms), the post-synaptic density of excitatory synapses (2/20) and vesicles/organelles (4/20). Additional significant GO Process and GO Function terms relate to synapse assembly, cell adhesion and the actin cytoskeleton. Ingenuity pathway analysis (IPA), a complementary method, identified similar terms, including terms predicting that the translocation will affect synapse assembly and function in mature neurons of the brain (Fig. 2d). IPA also found that the term ‘movement of organelle’ was significant (Fig. 2d). Given that the RNAseq analysis is unbiased, it is notable that *DISC1* regulates processes related to at least three of the major enriched categories; neurite extension;^10^ synaptic vesicle and mitochondrial trafficking^13,14^ and actin remodelling^11^. We therefore infer that these process-related gene expression changes are most likely due to DISC1 disruption.

While the translocation is unique to a single family, translocation carriers do not present unusual clinical symptoms outside the current diagnostic criteria, thus the consequences of the translocation may converge upon biological processes shared with other unrelated patients. In support of this, several of the differentially expressed genes in t(1;11) neurons correspond to genome-wide association study (GWAS) findings for schizophrenia^2,24^. Expression of 33 of the 348 genes from 25 of the 108 associated loci (hypergeometric probability *p* = 7E-6 indicates enrichment for loci containing at least one dysregulated gene) identified in the first study, and 37 of the 481 genes from 24 of the 145 loci (hypergeometric probability *p* = 0.001) identified in the second study is altered at the whole-gene and/or exon level in control versus translocation lines (Supplementary Table 1a, b). Of these dysregulated putative schizophrenia genes, changed expressions of *DRD2*, encoding the dopamine D2 receptor, a target of all antipsychotics in clinical use, and *PDE4B*, encoding a cAMP-degrading phosphodiesterase and known DISC1 interactor^25^, were confirmed by quantitative RT-PCR (Fig. 2e). Altered expression was also confirmed for genes encoding the historical candidate *NRG1*^26^ and its receptor *ERBB4* (Fig. 2e), both of which have previously been linked functionally to DISC1^27,28^. Expression of 12 of 52 genes encoding synaptic proteins that are targeted by recurrent CNVs in schizophrenia^3^ is also altered (hypergeometric probability *p* = 0.001, Supplementary Table 1a, b). Four of 70 genes at four of 44 depression-associated loci (9.1% of loci, no enrichment)^29^ identified through GWAS are also dysregulated (Supplementary Table 1a, b).

Finally, three of four recently identified putative modifier loci in the t(1;11) family^30^ are adjacent to the translocation breakpoints on chromosomes 1 or 11 (Supplementary Table 1f, g). Of the three genes of interest identified therein, *GRM5* is not dysregulated in the human cortical neurons (Fig. 2e, Supplementary Table 1a, b), *CAPN8* is expressed at very low, non-quantifiable levels and *CNTN5* is dysregulated according to the RNASeq analysis (*p* = 0.0008, Supplementary Table 1a, b). However, although quantitative RT-PCR also suggests increased *CNTN5* expression, this did not achieve statistical significance (Fig. 2e). The fourth locus, encompassing *PDE4D*, could not be examined because the cell lines used for RNASeq analysis and quantitative RT-PCR do not carry the required haplotype.

### A mutant mouse recapitulates the effect of the t(1;11) translocation upon *DISC1*

A mutant mouse was generated by deleting genomic DNA containing *Disc1* exons 9–13 and replacing it with human chromosome 11 genomic DNA containing relevant *DISC1FP1* exons (Fig. 1f). This mimics the gene fusion caused by the translocation on the derived chromosome 1^20^, thus we refer to the mutation as *Der1*.

Quantitative RT-PCR demonstrated that wild-type *Disc1* transcript expression is reduced by approximately half in whole brain isolated from adult heterozygous *Der1* (*Disc1*^*wt/Der1*^) mice in comparison with wild-type (*Disc1*^*wt/wt*^) mice (Fig. 1g). Full-length *Disc1* protein expression is detected at ~100 kDa, and is correspondingly reduced in *Disc1*^*wt/Der1*^ versus *Disc1*^*wt/wt*^ mice (Fig. 1h). As expected, wild-type *Disc1* transcripts and protein were undetectable in whole brain from homozygous (*Disc1*^*Der1/Der1*^) mice (data not shown, Fig. 1h). CP60/69 transcripts were detected using RT-PCR in whole brain from adult *Disc1*^*wt/Der1*^ and *Disc1*^*Der1/Der1*^ mice (Fig. 1i), and quantitative RT-PCR in *Disc1*^*Der1/Der1*^ mice demonstrated widespread expression of these transcripts (Supplementary Figure 3d). Quantitative analysis of wild-type *Disc1* expression in *Disc1*^*wt/wt*^ mice generated a similar pattern of expression to that of the chimeric transcripts (Supplementary Figure 3d), indicating that *Disc1* promoter activity is not greatly affected by the *Der1* mutation. Further characterisation of the *Der1* mutant mouse will be published elsewhere.

### Interaction between DISC1 and GluN1

**Fig. 3:**
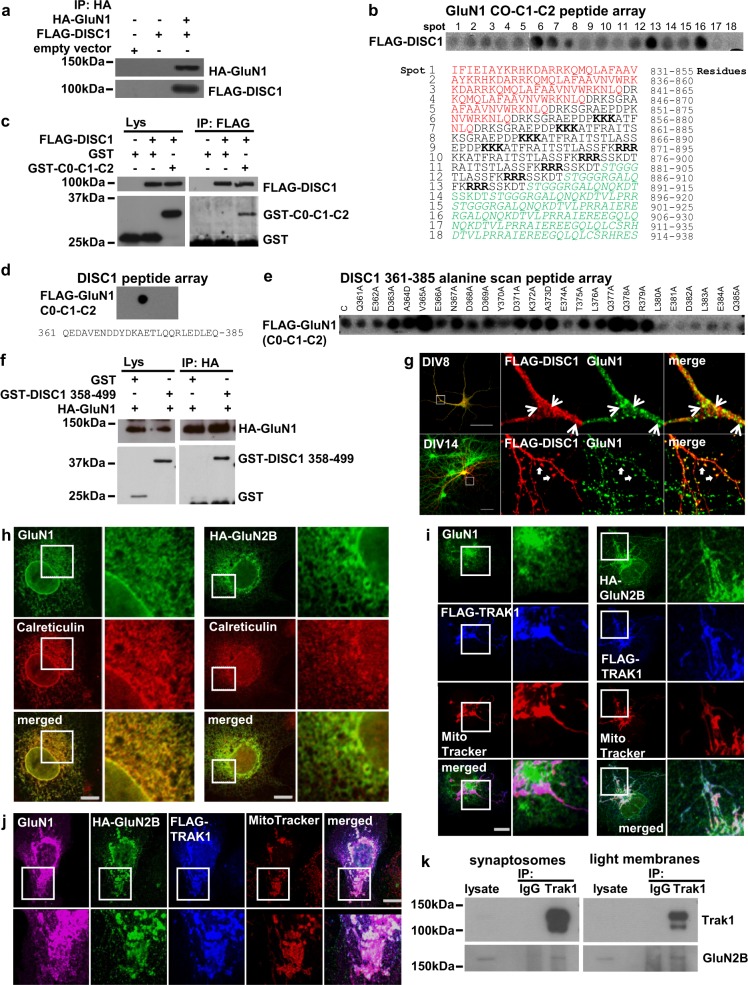
DISC1 interacts with GluN1 and TRAK1 associates with GluN2B. **a** HA–GluN1 co-immunoprecipitates FLAG-DISC1 from transfected COS7 cells. **b** GluN1 cytoplasmic tail (C0-C1-C2) peptide array hybridised with Flag-DISC1. Each spot represents a single peptide. Red, black and green texts mark the C0, C1 and C2 cassettes, respectively. ER retention signals are in bold. **c** FLAG-DISC1 co-immunoprecipitates GST-tagged C0-C1-C2. **d** DISC1 peptide array probed with FLAG-C0-C1-C2. **e** DISC1 361–385 GluN1 binding region alanine scan probed with FLAG-C0-C1-C2. C, unmutated peptide. **f** HA–GluN1 co-immunoprecipitates GST-DISC1 amino acids 358–499 from transfected COS7 cells. **g** Co-localisation of FLAG-DISC1 and endogenous GluN1 in cultured DIV8 and DIV14 hippocampal neurons. Arrowheads and arrows indicate example sites of co-localisation. **h** COS7 cells transfected with GluN1 or HA–GluN2B expression constructs were labelled with antibodies specific for GluN1 or HA, plus the ER marker Calreticulin. **i** COS7 cells co-transfected with FLAG-TRAK1 plus GluN1 (left) or HA–GluN2B (right) expression constructs were labelled using antibodies specific for GluN1 or HA, plus anti-FLAG and the mitochondrial dye Mitotracker CMXRos. **j** COS7 cells triple transfected with FLAG-TRAK1, GluN1 plus HA–GluN2B expression constructs were labelled using antibodies specific for GluN1, HA and FLAG, plus Mitotracker CMXRos. COS7 cells were used because they are ideal for exogenous protein expression due to their high transfection efficiency, large size and low profile, which facilitate co-immunoprecipitation and co-localisation studies to complement endogenous protein studies in neurons. **k** Trak1 immunoprecipitates GluN2B from adult mouse brain synaptosome and light membrane fractions. Scale bars, 50 μm in G, otherwise 20 μm; white boxes indicate enlarged areas

Immunoprecipitation experiments using endogenous brain proteins have previously demonstrated that GluN1 and Disc1 exist in shared complexes in vivo^31^. Here, using FLAG-DISC1 and HA-tagged GluN1, we confirmed that they can be co-immunoprecipitated using COS7 cells (Fig. 3a). We next used FLAG-DISC1 to probe peptide arrays of the GluN1 cytoplasmic tail, encompassing the alternatively spliced C0, C1 and C2 cassettes (Fig. 3b)^32^. FLAG-DISC1 was found to bind directly to GluN1 peptide spots 1–16, encompassing amino acids 831–930 and, most strongly, to three regions, within peptides 6–8, 13 and 16, corresponding to the C1, C2 and C0 cassettes. To confirm DISC1 binding to these regions, we demonstrated that FLAG-DISC1 efficiently co-immunoprecipitates GST-tagged GluN1 C0-C1-C2 from COS7 cell lysates (Fig. 3c). All known GluN1 isoforms contain at least one of these cassettes^32^ and thus can potentially interact with DISC1.

Reciprocal probing of a human DISC1 peptide array with the cytoplasmic tail of GluN1 fused to FLAG revealed a GluN1 contact site between amino acids 361–385 (Fig. 3d). To confirm this, an alanine scanning peptide array of DISC1 amino acids 361–385 was interrogated with the same GluN1 probe (Fig. 3e), revealing that binding to the wild-type sequence is substantially weakened by alanine conversion of any one of the amino acids E374, L380, E381, D382, L383, E384 and Q385. The DISC1 head domain therefore contacts GluN1 via a single site in which residues 380–385 are critical for binding. This binding site was independently confirmed by co-immunoprecipitating HA–GluN1 and GST-DISC1 amino acids 358–499 from COS7 cells (Fig. 3f). Moreover, exogenous DISC1 co-localises with endogenous GluN1 in cultured DIV8 and DIV14 hippocampal neurons (Fig. 3g). These observations indicate that DISC1 and the putative schizophrenia risk factor GluN1^3^ have the capacity to interact directly in vivo.

### TRAK1 complexes with GluN2B

Because DISC1 interacts with GluN1 and regulates cargo transport, we speculated that DISC1 and TRAK1 might be involved in NMDAR trafficking. To test this, we co-transfected COS7 cells with TRAK1 plus NMDAR subunit, GluN1 or GluN2B expression constructs. In the absence of TRAK1, both GluN1 and HA–GluN2B predominantly co-localise with the ER marker Calreticulin (Fig. 3h), while FLAG-TRAK1 is strongly targeted to the mitochondria, as expected^17^ (Fig. 3i). In co-transfected cells, there is no appreciable co-localisation between FLAG-TRAK1 and GluN1 (Fig. 3i), but in the presence of FLAG-TRAK1, HA–GluN2B redistributes from the ER to the mitochondria (Fig. 3i). Moreover, in triple-transfected cells, in the presence of HA–GluN2B plus FLAG-TRAK1, a substantial proportion of GluN1 localises in the mitochondria (Fig. 3j), indicating that assembled NMDAR can associate with TRAK1. We next immunoprecipitated Trak1 from mouse brain synaptosome and light membrane fractions, where both proteins are enriched^33^ (data not shown), and demonstrated co-precipitation of GluN2B (Fig. 3k, Supplementary Figure 4). The DISC1-associated trafficking factor Trak1 thus associates with the NMDAR GluN2B subunit in vivo.

DISC1, with TRAK1, is now well established as a regulator of neuronal intracellular trafficking^34^. The GluN1/ DISC1/TRAK1 and TRAK1/GluN2B associations therefore imply that DISC1 regulates the motility of GluN2B-containing NMDAR.

### DISC1 dysregulates dendritic GluN1–Dendra2 motility in mouse hippocampal neurons

**Fig. 4:**
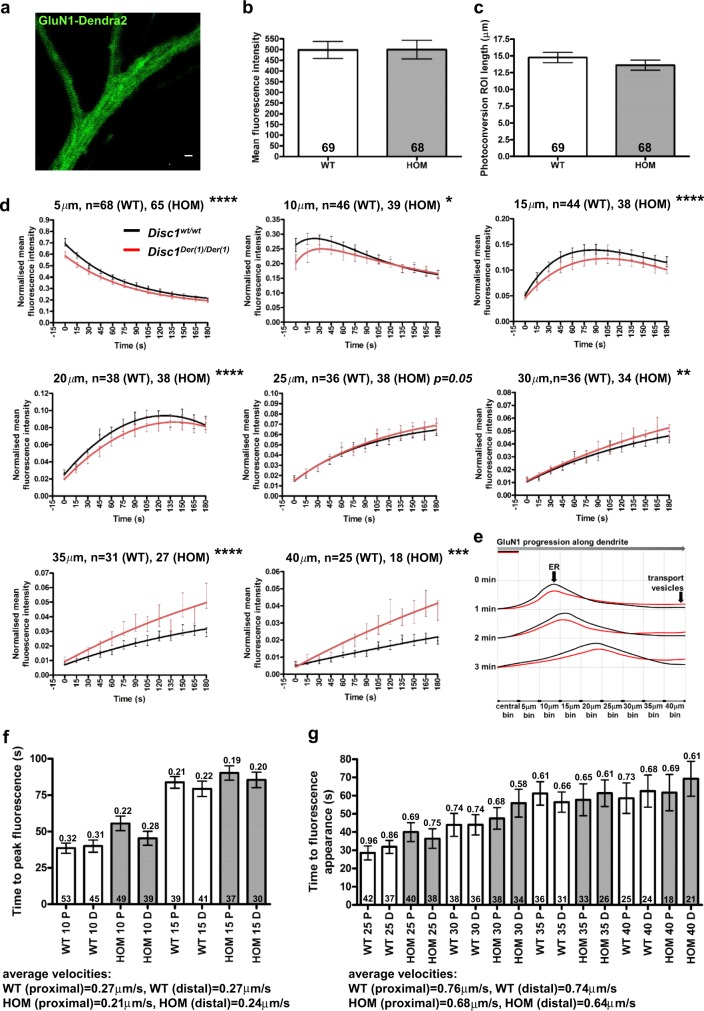
Altered distal dendritic NMDAR trafficking in *Disc1*^*Der1/Der1*^ hippocampal neurons. **a** Green Dendra2 fluorescence in dendrites of a DIV8 mouse hippocampal neuron transfected with GluN1–Dendra2 plus HA–GluN2B. Scale bar, 5 μm. **b** and **c** Mean red fluorescence intensity (**b**), or ROI dendritic length (**c**), in the central bin in *Disc1*^*wt/wt*^ and *Disc1*^*Der1/Der1*^ DIV8 neurons was equal at time zero following photoconversion. **d** Quantification of fluorescence intensity over time in successive 5 μm dendritic bins distal to the centre of the photoconversion ROI. Data analysed by timepoint-paired two-tailed *t* test. **e** Model of dendritic GluN1–Dendra2 motility. Photoconverted GluN1–Dendra2 progresses in a wave-like fashion, with the fastest and slowest moving GluN1–Dendra2 at the leading and trailing edges, respectively, and the bulk travelling as the ‘crest’. **f** Fluorescence peak velocity estimates for the 10-μm and 15-μm bins. Average time to peak fluorescence was converted to velocity, indicated above each bar. Average velocities were determined from the two bins. **g** Fast-moving GluN1–Dendra2 maximum velocity estimates for the 25-μm–40-μm bins. Average time to fluorescence appearance was converted to velocity, indicated above each bar. Average velocities were determined from the four bins. WT, *Disc1*^*wt/wt*^; HOM, *Disc1*^*Der1/Der1*^. Error bars represent SEM. ****p < 0.0001; ***p < 0.001; **p < 0.01; *p < 0.05; *n* indicated on graphs

To examine NMDAR motility in neurons, we fused the green fluorescent protein Dendra2 to GluN1, which is incorporated into all NMDAR. Dendritic GluN1–Dendra2 has a granular appearance similar to that of endogenous GluN1 (Fig. 4a, Supplementary Figure 5a)^35^. Dendra2 can be stably photoconverted to red fluorescence, enabling tracking of a specific red GluN1 population (Supplementary Video). Dendra2 fusion to the GluN1 C terminus does not interfere with the receptor assembly or trafficking to synapses because GluN1–Dendra2 co-localises with the post-synaptic density marker PSD95 within dendritic spines (Supplementary Figure 5b). This can only occur following proper receptor assembly^5^.

Bulk GluN1–Dendra2 movement was quantified in cultured wild-type hippocampal neurons expressing exogenous human DISC1 at days in vitro 8 (DIV8), an age at which the dendritic arbour is suitably developed, but not too complex. Following GluN1–Dendra2 photoconversion, red fluorescence bidirectional spreading was imaged and analysed every 15 s for a total of 2 min in successive 5-μm bins along the dendrites (Supplementary Figure 5c-n).

We examined the effects of wild-type DISC1 and a change of arginine to tryptophan at position 37 (37W) in DISC1 (Supplementary Figures 6a–f, 7a, b). This sequence variant has been reported in four individuals, all with a diagnosis of psychiatric illness^36,37^, and dysregulates DISC1/TRAK1 association^17^. Wild-type DISC1 overexpression increases distal-moving red fluorescence in the 20−35-μm bins (Supplementary Figure 6c, d), while in the proximal direction, it reduces red fluorescence intensity in all bins (Supplementary Figure 7a, b). In contrast, DISC1–37W overexpression increases and decreases distal-moving red fluorescence in the 5−15-μm and 25−35-μm bins, respectively (Supplementary Figure 6c, d), while proximal-moving red fluorescence increases in the 5–20-μm bins (Supplementary Figure 7a, b).

These data are consistent with the existence of at least two classes of distal-moving dendritic GluN1–Dendra2 in young hippocampal neurons that are differentially influenced by DISC1: (1) a slow-moving population in the 5–15/20-μm bins (average velocity = 0.2 μm/s in empty vector-transfected neurons, Supplementary Figures 6d, e, 7b), representing the majority of the GluN1–Dendra2 and (2) a fast-moving population in the 25–35-μm bins (average velocity = 0.82 μm/s in empty vector-transfected neurons, Supplementary Figures 6d, f, 7b).

These populations represent the trailing edge plus ‘crest’ and the leading edge, respectively, of a wave of motile GluN1–Dendra2 (Supplementary Figures 6d, 7b). Our evidence for these populations aligns well with estimates of 0.2–0.3μm/s for the movement of dendritic ER vesicles^38^ and with estimates of 0.76 μm/s for the motility rate of assembled NMDAR-containing vesicles undergoing fast active transport in dendrites^39^, thus the slow and fast populations likely represent the ER GluN1 pool and NMDAR-containing vesicles undergoing active transport, respectively.

### Altered dendritic NMDAR motility in *Der1* mouse hippocampal neurons

We next quantified bulk GluN1–Dendra2 movement within neuronal dendrites from *Disc1*^*wt/wt*^ and *Disc1*^*Der1/Der1*^ mice using the same method (Fig. 4b–g, Supplementary Figure 7c, d). The same two pools of GluN1 were detected, with the distal-moving ER and transport vesicle pools, respectively, decreased and increased in *Disc1*^*Der1/Der1*^ mutant neurons (Fig. 4d, e). A similar, but less marked, pattern of proximal GluN1–Dendra2 motility was observed (Supplementary Figure 7c, d).

### Altered dendritic NMDAR surface distribution in *Der1* mouse hippocampal neurons

The NMDAR trafficking data indicate that DISC1 modulates NMDAR motility and consequently predict dysregulated cell surface NMDAR expression in neurons cultured from the *Der1* mutant mouse. To test this, we quantified the NMDAR cell surface expression in DIV21 cultured *Disc1*^*wt/wt*^, *Disc1*^*wt/Der1*^ and *Disc1*^*Der1/Der1*^ hippocampal neurons, an age at which neurons have matured and developed synapses. Cell surface expression of the NMDAR subunits GluN1, GluN2A and GluN2B and of the post-synaptic density marker PSD95 and βIII-tubulin (Tuj1) was imaged at high resolution by three-dimensional structured illumination microscopy (3D-SIM, Supplementary Figure 8a), which achieved a lateral image resolution of 180 nm for the NMDAR subunits and 120 nm for PSD95.

**Fig. 5:**
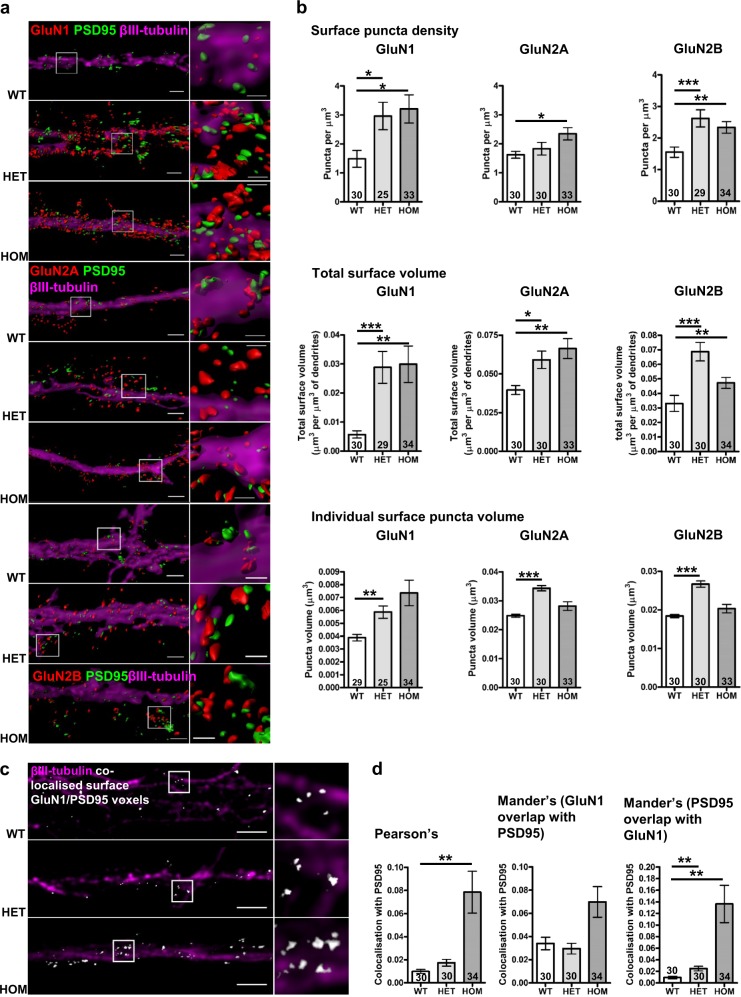
Altered dendritic NMDAR surface expression and GluN1 localisation to the post-synaptic density in *Disc1*^*wt/Der1*^ and *Disc1*^*Der1/Der1*^ hippocampal neurons. **a** Objects in 3D-SIM images were visualised using the Imaris Isosurface tool. Touching objects are separated with boundary lines between touching objects visible in the enlarged images. Scale bars, 2 μm in the full-size images, 0.6 μm in enlarged insets indicated by white boxes (**b**) GluN1, GluN2A or GluN2B surface puncta density, total surface volume and individual surface puncta volume (all normalised to dendritic segment volume) from 3D reconstructions of primary dendrite segments of cultured DIV21 hippocampal neurons. Data analysed by Kruskal–Wallis (puncta density *p* = 0.02, *p* = 0.03, *p* = 0.0004, total volume *p* = 0.0025, *p* = 0.4, *p* = 0.003, puncta volume *p* = 0.003, *p* < 0.0001, *p* < 0.0001 for GluN1, GluN2A and GluN2B respectively) followed by Dunn’s multiple comparison test. **c** Reconstructed 3D-SIM images of dendrites. Co-localised voxels, which contain signals from both PSD95 and surface-expressed GluN1 are shown in white. Scale bars, 3 μm. **d** GluN1 co-localisation with the PSD95 was evaluated on three measures. Pearson’s and Mander’s coefficients, respectively, indicate the overall correlation of each signal or amount of GluN1 signal co-localised with PSD95 signal, and vice versa. Data were analysed by Kruskal–Wallis (*p* = 0.004, *p* = 0.08, *p* = 0.001 for Pearson’s, Mander’s M1 and Mander’s M2), followed by Dunn’s multiple comparison test. WT, *Disc1*^*wt/wt*^; HET, *Disc1*^*wt/Der1*^; HOM and *Disc1*^*Der1/Der1*^. Error bars represent SEM. ***p < 0.001; **p < 0.01 and *p < 0.05; *n* indicated on graphs

3D reconstructions of βIII-tubulin and GluN1, GluN2A and GluN2B were used to estimate dendritic volume and the number and volume of individual surface puncta of each NMDAR subunit, respectively (Fig. 5a, b, Supplementary Table 2). This analysis demonstrated increased density of GluN1 and GluN2B puncta in *Disc1*^*wt/Der1*^ and *Disc1*^*Der1/Der1*^ mutant neurons, while GluN2A puncta density is increased only in *Disc1*^*Der1/Der1*^ mutant neurons. Total surface expression is correspondingly increased in *Disc1*^*wt/Der1*^ and *Disc1*^*Der1/Der1*^ mutant neurons. Average puncta volumes are increased for all three NMDAR subunits, but only in *Disc1*^*wt/Der1*^ neurons, suggesting that NMDAR form larger clusters on the cell surface in heterozygous neurons. This observation supports the existence of putative aberrant proteins encoded by the chimeric CP60/69 transcripts. These proteins retain a region that is sufficient for self-association^40^, so there is potential for dominant-negative effects due to the formation of multimers consisting of wild-type and mutant protein, a situation that occurs only in heterozygous cells.

At the neuronal cell surface NMDAR are present at synaptic, perisynaptic and extrasynaptic sites^5^, thus only a proportion of receptors co-localise with PSD95 at synapses. Applying Pearson’s co-localisation coefficient, which quantifies the overall correlation between two fluorescent signals, indicates that PSD95 co-localisation with GluN1 is substantially increased in *Disc1*^*Der1/Der1*^ mutant neurons (Fig. 5c, d). However, synaptic localisation of GluN2A and GluN2B is not greatly altered (Supplementary Figure 9a, b). Since GluN1 subunits cannot be transported to synapses unless they have assembled with other subunit types, we suggest that this discrepancy is related to the presence of two GluN1 subunits in every NMDAR, with variable inclusion of other subunit types, resulting in a clear effect for the former, but not for the latter. If correct, the GluN1/PSD95 co-localisation data indicate that NMDAR are more abundant at synapses in the *Disc1*^*Der1/Der1*^ mutant neurons.

Hippocampus protein extract immunoblotting demonstrated that total expression of GluN1, GluN2A and GluN2B is unaltered by mutation in adult *Disc1*^*wt/Der1*^ or *Disc1*^*Der1/Der1*^ mice (Supplementary Figure 9c), confirming that surface expression changes are due to altered NMDA receptor subunit distribution.

### Altered distribution of PSD95 in *Der1* mouse hippocampal neurons

PSD95 is one of the most abundant molecular scaffolds at the post-synaptic density of excitatory synapses. It has many functions including organisation of the post-synaptic density, NMDAR stabilisation at synapses, assembly of NMDAR-associated signalling complexes and recruitment of AMPA receptors (AMPAR), the major excitatory receptors in the brain^41^. PSD95 is thus a major determinant of excitatory synapse function and neurotransmission. Expression of PSD95 was therefore quantified in the imaged set of hippocampal neurons to determine whether synapse size and structure is also affected by *Der1* mutation.

**Fig. 6:**
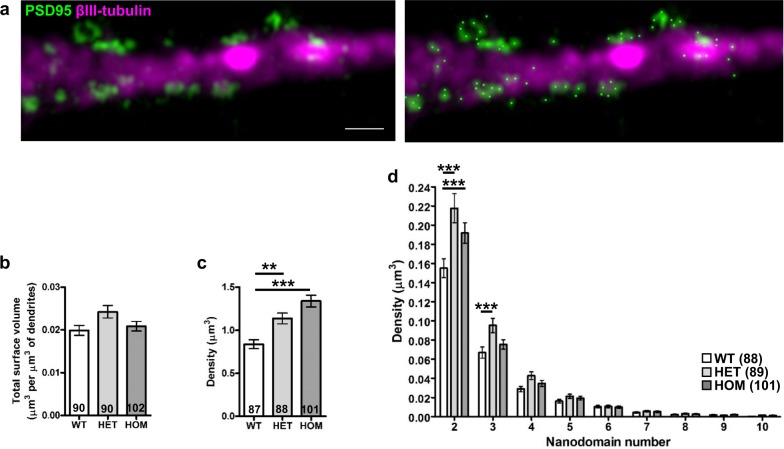
Altered PSD95 distribution in *Disc1*^*wt*/*Der1*^ and *Disc1*^*Der1*/*Der1*^ hippocampal neurons. **a** Left, raw 3D-SIM image of a dendrite with PSD95 clusters and nanodomains. Right, the same image with nanodomain centres (bright green spots) identified using Imaris. Scale bar, 1 μm. **b** PSD95 total volume normalised to dendritic segment volume. Data analysed by Kruskal–Wallis (not significant). **c** Quantification of single PSD95 nanodomains. Data analysed by one-way ANOVA (*p* < 0.0001). **d** Quantification of PSD95 nanodomain number per cluster. Data analysed by two-way ANOVA which found a significant interaction between genotype and nanodomain number per cluster (*p* < 0.0001). WT, *Disc1*^*wt/wt*^; HET, *Disc1*^*wt/Der1*^; HOM and *Disc1*^*Der1/Der1*^. Error bars represent SEM. ***p < 0.001 and **p < 0.01; *n* indicated on graphs

Super-resolution microscopy has demonstrated the existence of varying numbers of PSD95 puncta (nanodomains) at the post-synaptic density^42–44^. Most synapses have a single PSD95 nanodomain, but some have multiples^42–44^. Using 3D-SIM, PSD95 is visible as a combination of single spots and distinct clusters of multiple puncta (Fig. 6a, Supplementary Figure 8), indicating that this super-resolution technique is able to resolve PSD95 nanodomains within clusters at the post-synaptic density. Total dendritic PSD95 volume is not affected by the *Der1* mutation (Fig. 6b), however its distribution is altered. Analysis of the number of nanodomains per cluster (Supplementary Figure 8b–f) found that *Disc1*^*wt/Der1*^ and *Disc1*^*Der1/Der1*^ neurons have an increased density of single nanodomains (Fig. 6c), and of small nanodomain clusters of 2–3 (Fig. 6d).

## Discussion

Recent large-scale GWAS and CNV studies implicate excitatory synapses, NMDAR subunits GluN1 and GluN2A, and synaptic plasticity as causal factors in schizophrenia^2–4,24^. We show here that multiple genes identified in these studies are differentially expressed in human neurons carrying a t(1;11) linked to major mental illness. Moreover, the gene *DISC1*, disrupted by the t(1;11), encodes a direct interactor of the NMDAR obligatory GluN1 subunit. This convergence of the t(1;11) with schizophrenia risk genes indicates that the t(1;11) is a trigger for disease pathways, which is shared with unrelated schizophrenia patients. Affective disorders are also prevalent in t(1;11) carriers, and we found differential expression of putative depression risk factors in the t(1;11)-carrying human neurons, although not as strikingly as for schizophrenia.

We demonstrate that DISC1 regulates NMDAR motility within dendrites, a process that is critical for controlling cell surface and synaptic NMDAR expression. NMDAR motility is influenced by a DISC1–37W mutation^36,37^, previously shown to affect mitochondrial motility^17^, and by the mouse *Der1* mutation which accurately models the effect of the t(1;11) upon DISC1 expression. DISC1, DISC1–37W and the *Der1* mutation influence the size of the GluN1 ER pool, indicating, in the absence of altered NMDAR subunit expression, that DISC1 regulates the NMDAR ER-exit. Indeed DISC1 is ER-associated^16^, and may directly impact NMDAR ER-exit because the binding site for DISC1 on GluN1 encompasses two ER retention signals (Fig. 3b)^5^. However, a simple model whereby increased/decreased ER-exit leads to, respectively, decreased/increased transport pool size cannot fully explain the effects of DISC1 upon NMDAR motility due to the lack of an overt relationship between the observed sizes of the ER and active transport pools. We therefore propose that DISC1 influences the active transport pool independently of ER-exit via TRAK1, which associates with the NMDAR subunit GluN2B, and its role as a regulator of neuronal cargo trafficking^34^.

Mouse neurons expressing DISC1–37W exhibit a decreased pool of NMDAR undergoing fast active transport, while *Der1* mutant mouse neurons exhibit an increased pool. Since the 37 W variant has to date been found only in psychiatric patients, this suggests that dysregulation of this pool, up or down, is deleterious. Upregulation of the pool in *Der1* mutant mouse neurons correlates strikingly with the augmented density and overall volume of cell surface NMDAR clusters, suggesting that increased trafficking/membrane insertion largely account for the greater surface NMDAR volume. NMDAR are constantly exchanged between synaptic and extrasynaptic sites^45^, with synaptic insertion events believed to result from stochastic post-synaptic density-mediated capture of receptors undergoing lateral diffusion within the cell membrane^46^. Consequently, the elevated NMDAR surface volume in *Der1* mutant mouse neurons likely increases the probability of their diffusion to, and insertion into, synapses, thus explaining the increased synaptic localisation of GluN1.

In addition to the NMDAR abnormalities, the post-synaptic density scaffold PSD95 is aberrantly distributed in *Der1* mutant mouse neurons. Post-synaptic density size at excitatory synapses is directly related to the number of PSD95 nanodomains present^43^ and to AMPAR expression and synapse size/strength^41^. Moreover, the number of PSD95 nanodomains at a synapse is proposed to be directly related to the number of AMPAR at a synapse^42,44,47^. Synaptic PSD95 nanodomain number therefore likely predicts the synaptic AMPAR expression and in turn, synapse strength, which is determined by the number of AMPAR present. On this basis, the *Der1* mutant mouse neurons are predicted to possess fewer synaptic AMPARs and weaker synapses.

AMPAR-induced depolarisation initiates NMDAR-dependent synaptic plasticity and long-term potentiation via molecular pathways involving signalling molecules such as CAMKII, PKA, PDE4 and the transcription factor CREB^48–52^. This leads to increased synaptic AMPAR expression and dendritic spine volume through actin remodelling, and thus greater synapse strength and size^53,54^. Various DISC1 mutant mice exhibit synaptic plasticity defects^34^ and in some cases involvement has been demonstrated of PDE4, which interacts with and is regulated by DISC1^25^ or CREB^34^. Moreover, DISC1 is known to regulate the AMPAR subunit expression and dendritic spine size/density downstream of NMDA receptors via actin remodelling^11,55^, and could therefore control plasticity through these mechanisms. DISC1 is also reported to regulate the NMDAR activity through PDE4-mediated activation of CREB-dependent GluN2A expression^12^, although we found no evidence of this mechanism in *Der1* mice since hippocampal GluN2A levels are unaltered. DISC1 may therefore regulate synaptic plasticity at multiple levels downstream of NMDAR, and it is notable that the gene expression changes in the human neurons derived from t(1;11) carriers, including altered expression of *PDE4B* and genes required for actin cytoskeleton remodelling, are a good fit with these processes. Crucially, however, our data indicate that DISC1 has the potential to modulate the triggering of plasticity itself, through effects upon synapse strength and via direct interaction with GluN1, regulation of NMDAR dynamics and thus control of their synaptic expression and availability to initiate plasticity. Synaptic plasticity underlies cognition which is characteristically impaired in schizophrenia in particular, but also in affective disorders. Our findings thus point towards a pathway that is of direct relevance to a core feature of psychiatric disorders in t(1;11) carriers and to major mental illness in general.

Four potential genetic modifiers have recently been identified in the t(1;11) family^30^. Of the genes-of-interest identified at these loci, only *GRM5* and *CNTN5* could be meaningfully examined using the selected panel of t(1;11) family neurons and although abundantly expressed, neither was found to be dysregulated. Nor were any deleterious sequence variants that would affect protein function discovered in these genes^30^, although potential expression quantitative trait loci (eQTLs) that could affect the *GRM5* expression levels were previously identified within the modifier^30^. Such eQTLs may therefore act upon *GRM5* in a cell type-specific manner. The *Der1* mouse mimics disruption of *DISC1* by the t(1;11), isolating this event from any effects of the putative modifiers. We therefore propose that the deleterious effects of DISC1 disruption upon excitatory synapses, identified here using the *Der1* mutant mouse, contribute substantially to risk of major mental illness in t(1;11) carriers, but can be influenced by the genetic modifiers and/or environmental factors, leading to differing outcomes. This is consistent with a previous observation that, irrespective of diagnosis, all tested t(1;11) carriers exhibit an abnormal schizophrenia endophenotype, the P300 event-related potential^6^, and that P300 abnormalities are linked to NMDAR dysfunction^56^.

In conclusion, the *Der1* mutation-induced NMDAR trafficking and synapse abnormalities described here converge upon themes of dysfunctional NMDAR, excitatory synapses and plasticity that are emerging from schizophrenia and depression GWAS and CNV data. The t(1;11) thus provides a mechanistic window into the biochemical basis of these disorders, which links to genetic findings and highlights new ways to consider therapeutic intervention.

## Electronic supplementary material

Supplementary video

SI word file

Data Set 1
