# Supplementary material for: DISC1 regulates N-methyl-D-aspartate receptor dynamics: abnormalities induced by a Disc1 mutation modelling a translocation linked to major mental illness
Source: Transl Psychiatry. 2018 Sep 6;8:184. doi: 10.1038/s41398-018-0228-1 (PMC6127284; doi:10.1038/s41398-018-0228-1)
Supplement: Supplementary file 2 — SI word file [file 41398_2018_228_MOESM2_ESM.docx]

# Supplementary information

**DISC1 regulates N-Methyl-D-Aspartate receptor dynamics: Abnormalities induced by a *Disc1* mutation modelling a translocation linked to major mental illness**

Elise L.V. Malavasi1, Kyriakos D. Economides2*, Ellen Grünewald1*, Paraskevi Makedonopoulou1*, Philippe Gautier4, Shaun Mackie1, Laura C. Murphy1, Hannah Murdoch5, Darragh Crummie1, Fumiaki Ogawa1, Daniel L. McCartney1, Shane T. O’Sullivan1, Karen Burr6, Helen S. Torrance1, Jonathan Phillips1, Marion Bonneau1, Susan M. Anderson1, Paul Perry4, Matthew Pearson4, Costas Constantinides1, Hazel Davidson-Smith1, Mostafa Kabiri8, Barbara Duff7, Mandy Johnstone1,7, H. Greg Polites9, Stephen M. Lawrie7, Douglas Blackwood7, Colin A. Semple4, Kathryn L. Evans1, Michel Didier10, Siddharthan Chandran6, Andrew M. McIntosh7, David J. Price11, Miles D. Houslay12, David J. Porteous1 and J. Kirsty Millar1**

1Centre for Genomic and Experimental Medicine, MRC Institute of Genetics and Molecular Medicine at the University of Edinburgh, UK, 2Xtuit Pharmaceuticals, Waltham MA, USA, 4MRC Human Genetics Unit, MRC Institute of Genetics and Molecular Medicine at the University of Edinburgh, UK, 5Molecular Pharmacology Group, Wolfson Building, Institute of Neuroscience and Psychology, The University of Glasgow, University Avenue, Glasgow, UK, 6Centre for Regenerative Medicine, The University of Edinburgh, UK, 7Division of Psychiatry, The University of Edinburgh, UK, 8Translational In Vivo Models at Sanofi, Frankfurt, Germany, 9Regeneron Pharmaceuticals, Tarrytown NY, USA, 10Translational Sciences at Sanofi, Chilly-Mazarin, France, 11Centre for Integrative Physiology, The University of Edinburgh, UK, 12School of Cancer and Pharmaceutical Sciences, King's College London, UK

* These authors contributed equally

**Corresponding author

Centre for Genomic and Experimental Medicine, MRC Institute of Genetics and Molecular Medicine at the University of Edinburgh, Crewe Road, Edinburgh EH4 2XU, UK

Tel: +44 (0)131 651 8732

Fax: +44 (0)131 651 1059

Email: [kirsty.millar@igmm.ed.ac.uk](mailto:kirsty.millar@ed.ac.uk)

# MATERIALS AND METHODS

**Generation of induced pluripotent stem cells (IPSC) from dermal fibroblasts**

Ethical consent relating to the translocation family is as follows: Prior to 2014, Lothian Research and Development (2011/P/PSY/09), Scotland A Research Ethics Committee (09/MRE00/81); 2014 onwards, Lothian Research and Development (2014/0303), Scotland

A Research Ethics Committee (14/SS/0039). Fibroblasts were cultured in DMEM with 10% FBS (all media and supplements from Life Technologies unless stated otherwise) at 37oC with 5 % CO2. Fibroblasts were reprogrammed by non-integrating methods, using episomal plasmids1. For episomal reprogramming of some lines we used a protocol adapted by Tilo Kunath, (University of Edinburgh) and Roslin Cells (roslincells.com). Other lines were reprogrammed by Roslin Cells. Plasmids incorporating Oct3/4, shRNA to p53, SOX2, KLF4, L-MYC and LIN28 were electroporated into fibroblasts using Nucleofection (Amaxa, Lonza). The episomal plasmids pCXLE-hOCT3/4-shp53-F (for OCT3/4 and p53 knockdown), pCXLE-hSK (for SOX2 and KLF4) and pCXLE-hUL (for L-MYC and LIN28) were a gift from Shinya Yamanaka. (These correspond to Addgene plasmids 27077, 27078, 27080). 5 x 105 fibroblasts were transfected with 1.7ug of pCXLE-hOCT3/4-shp53-F, 1.6ug of pCXLE-hSK and 1.7ug of pCXLE-hUL using the NHDF Nucleofector kit and Amaxa Nucleofector protocol U-023 (1,650 V, 10 ms, 3 time pulses), according to the manufacturer’s instructions. Cells were then seeded into one well of a gelatin-coated 6 well tissue culture grade plate in Opti- MEM (ThermoFisher Scientific) supplemented with 10% FCS and 1% Antibiotic Antimycotic Solution (Life Technologies 15240062). All stages of cells were maintained in media supplemented with Antibiotic Antimycotic Solution thereafter. The medium was replaced every 2-3 days before cells were replated into a 10cm Vitronectin/Geltrex (ThermoFisher Scientific) or Matrigel (Life Technologies)-coated tissue culture grade dish after 6-7 days.

The following day the medium was changed to Essential 6 medium (ThermoFisher Scientific) with added 100 ng/ml bFGF (Peprotech). The medium was changed every 2 days until colonies were ready to be picked, at approximately day 25-30. Individual colonies were

picked and expanded into 12, then 6, well Vitronectin/Geltrex or Matrigel-coated tissue culture grade plates in Essential 8 medium (ThermoFisher Scientific) with daily medium changes. Cells were passaged using 0.5mM EDTA in PBS. IPSCs were generated and cultured at 37oC, 5% CO2 and 21% O2. Quality control of IPSC lines was performed after clonal passage 10.

Pluripotency of IPSC lines was assessed using markers SSEA-1 PE, SSEA-4- AlexaFluor647 and Oct3/4 PerCP-Cy5.5 and isotype controls using the BD Stemflow Human and Mouse Pluripotent Stem Cell Analysis Kit (BD Biosciences, 560477) according to the manufacturer’s instructions, or with SSEA-1-APC (301907), SSEA-4-FITC (330409), TRA-1- 60-PE (330609), TRA-1-81-PE (330707) and isotype controls (all BioLegend) as follows: IPSCs were dissociated using StemPro Accutase (Life Technologies) and washed with Essential 8 medium. 1x105 cells were incubated with antibodies in 2% Fetal Bovine Serum in PBS for 1 hour on ice. Cells were washed once with 2% BSA/PBS, centrifuged at 200 x g for 5 min and resuspended in 200 ul 2% BSA/PBS. FACS analysis was performed on single cell suspensions using a FACS Aria cell sorter (BD Biosciences). Data were analysed using FlowJo v10 software.

EBNA-1 primer sequences and amplification protocol were taken from the Epi5 Episomal iPSC Reprogramming Kit (Life Technologies, A15960). Genomic DNA was extracted from IPSCs using the DNeasy kit (Qiagen). Cells that had not been in contact with episomes, were used as negative controls. Positive controls were low passage IPSC lines where episomes were still present. A non-template control (NTC) was also used. IPSC lines were only taken forward once episomal clearance had been confirmed by this method (data not shown).

The human Cytoscan 750 K Array (Affymetrix) was used to identify genomic abnormalities in the IPSC lines. The array consists of 550,000 unique non-polymorphic probes and 200,000 SNPs for accurate genotyping. Genomic DNA was extracted from IPSC clones using the DNeasy kit (Qiagen) according to manufacturer’s instructions. Samples were sent to the NHS Cytogenetics Laboratory (Western General Hospital, Edinburgh) for

processing of the arrays. Chromosome analysis was performed using Chromosome Analysis Suite version 2.0 (Affymetrix). Copy number, breakpoints and Loss Of Heterozygosity (LOH) regions were determined using the models and algorithms incorporated within the software package. To exclude possible false positives due to inherent microarray noise the CNV threshold of gains and losses for inclusion in analyses was 10 kilobase pairs (kbp) and 10 consecutive markers. IPSC lines with deletions or duplications greater than 5 MB, the limit typically applied by G-banding, were excluded from further studies.

# NPC culture and neuronal differentiation

IPSCs were converted into neuroectoderm by dual-SMAD signalling inhibition2. Long-term anterior neural precursor cells were generated and maintained under physiological normoxia (3% O2) and in the absence of EGF3. NPCs were cultured at 37oC, with 5% CO2 and 3% O2 on Matrigel (Life Technologies)-coated 6 well tissue culture grade plates in Advanced DMEM/F-12 (Life Technologies) with 1% Glutamax-1 (Life Technologies), 1% N2 supplement (Life Technologies), 0.1% B27 supplement (Life Technologies), 10 ng/ml bFGF (PeproTech) and 1% antibiotic/antimycotic solution (Life Technologies). NPCs were maintained up to passage 30 with feeding every 2-3 days and weekly passages using StemPro Accutase (Life Technologies). All NPC lines were tested every week for mycoplasma infection. For differentiation into cortical forebrain-like neurons3, NPCs were plated into Matrigel (Life Technologies)- and Laminin (Sigma-Aldrich)-coated 12 well tissue culture grade plates in Advanced DMEM/F-12 with 0.5% Glutamax-1, 0.5% N2 supplement, 0.2% B27 supplement, 2 μg/ml Heparin and 1% antibiotic/antimycotic solution (Life Technologies). Neurons were maintained for 5 weeks with feeding as necessary. During weeks 2 and 3 the neuronal differentiation medium was supplemented with Forskolin (Tocris Bioscience). During weeks 4 and 5 the Forskolin was removed, and the medium was supplemented with BDNF (Life Technologies) plus GDNF (Life Technologies) to 5ng/ml each.

# Mouse generation and colony maintenance

VelociMouse® technology (Regeneron)4 was used to target embryonic stem cells and microinject them into mouse embryos. In brief, F1H4 (129S6SvEv/C57BL6F1) embryonic stem cells were electroporated with the linearized vector construct and positive clones were microinjected into 8-cell stage mouse C57BL6 embryos. Microinjected embryos were transferred to uteri of pseudopregnant recipient females, weaned pups were scored, and high percentage chimera males were selected for mating with flp-positive C57BL6 females to remove the selection cassette, to prove germ-line transmission, and to generate F1 animals for further breeding.

Because there is already a mutation (25bp deletion) at the *Disc1* allele in exon 6 in the 129/Sv strain which causes a truncation of *Disc15*, F1 progeny were generated and a PCR assay which distinguishes the C57BL/6 allele versus the 129/Sv allele was employed to determine which F0 mice were correctly targeted to the C57BL/6 locus (Supplementary Figure 10). Mice which carried the translocation on the C57BL/6 allele were then crossed to CMV-Cre mice to remove the Neo cassette via Cre-mediated recombination at the flanking loxP sites. Genotyping results were confirmed by Loss-of-Native-Allele assay.

The exclusion of the differentially spliced *DISC1FP1* exon 3a6 that is present in a minority of transcripts (www.genome.ucsc.edu) precludes production of transcripts encoding CP1. The exclusion of the differentially spliced *DISC1FP1* exon 7b does not affect the potential production of CP60/69 proteins since the stop codon in chimeric transcripts encoding these proteins occurs in exon 66. Since the *Disc1* allele was modified on a mixed background of 129 and C57BL/6J, a congenic breeding strategy was adopted to purify the strain background. Following repeated crossing to C57BL/6J mice, genotyping of polymorphic markers carried out by the Jackson Laboratory found the mice to be >99.5% C57BL/6J. These mice were then mated to C57BL/6J for one final round and the progeny used for subsequent experiments. Mice were housed in the Biomedical Research Facility at the University of Edinburgh. All mice were maintained in accordance with Home Office

regulations, and all protocols were approved by the local ethics committee of the University of Edinburgh.

# Expression constructs

Constructs pCB6-HA-GluN2B7, expressing full-length rat GluN2B tagged at the N terminus with the HA epitope, pCB6-GluN17 and a plasmid expressing HA-GluN1 were gifts from William Green (University of Chicago). A GluN1-YFP expression construct was a gift from Seth Grant (University of Edinburgh).

Full length GluN1 and the C-terminal tail of GluN1 (C0-C1-C2) were subcloned into pCMV4A (Agilent) to generate FLAG-tagged constructs using the following Not1-Sal1 primer combinations: gatcgcggccgcaccatgagcaccatgcacctgctgacattcgcc, gatcgtcgacgctctccctatgacgggaacac and gatcgcggccgccaccatggagatcgcctacaagcgacacaag, gatcgtcgacgctctccctatgacgggaacacagctg. The GluN1 C0-C1-C2 sequence was subcloned into the BamH1-Not1 site of pEBG-2T to produce a GST fusion.

A DISC1 open reading frame consisting of amino acids 358-499 was inserted into the BamH1-Not1 sites of pEBG to generate pEBG GST-DISC1 358-499. The full-length DISC1 open reading frame was inserted into the same plasmid to generate pEBG GST-DISC1.

Plasmid pGluN1-Dendra2, expressing full-length human GluN1 (transcript variant GluN1-1a) tagged at the COOH terminus with Dendra2, was generated as follows. Dendra2 coding sequence was amplified from pDendra2-N (Clontech) using primer pair gatcgcggccgctcgagatgaacaccccgggaattaacc and

gatcggccggccttaccacacctggctggg and sub-cloned between the NotI and FseI sites of construct pCMV6-AC-NR1 (GluN1)-GFP (Origene RG216458).

Generation of pcDNA4/TO constructs expressing FLAG-TRAK1, FLAG-DISC1, FLAG-DISC1-37W and a corresponding empty vector has been described previously8, 9. All expression constructs were verified by sequencing.

# RNA isolation and cDNA synthesis

Total RNA was extracted using the RNeasy mini Kit (Qiagen), according to the manufacturer’s instructions. Genomic DNA was removed prior to cDNA synthesis using the TURBO DNA-free Kit (Thermo Fisher Scientific) and cDNA synthesis was performed using Dynamo cDNA synthesis kit (Thermo Scientific).

# RT-PCR

RT-PCR was performed using the Titanium Taq PCR Kit (Clontech). Each reaction contained cDNA template, 2.5μl PCR buffer, 0.5μl dNTPs, 1μl of both primers (10μM stock) and 0.5μl enzyme in a total volume of 25μl. Non-template controls were created by replacing the cDNA template with water.

# Quantitative RT-PCR

Quantitative Real-Time PCR was performed in 384-well plates using Power SYBR green PCR Master Mix (Applied Biosystems) on the 7900 HT sequence Detention System (Applied Biosystems). Non-template and minus reverse transcriptase controls were included in all experiments with three technical replicates for all samples. To control for inter-plate variation a calibrator sample was included on every plate for normalisation purposes. For quantification of human DISC1, housekeeping gene stability was assessed across samples taken from NPCs through to five week neurons for several genes using geNorm (genorm.cmgg.be/). *GAPDH* and *ACTB* were subsequently selected as the most stable housekeeping genes for use in quantitative RT-PCR in these samples. For quantification of mouse *Disc1*, *Cyclophilin* and *Hmbs* were found to be stable in the mouse brain samples analysed. *ACTB* was used as a reference gene for neuron RNASeq follow-up. Primer pairs were optimised for amplification efficiency and their specificity confirmed by PCR product sequencing. Melting curve analysis was carried out for each primer pair to optimise amplification conditions and confirm amplification specificity. PCR efficiency was assessed by running standard curves using serial dilutions of NPC or mouse brain samples for human

and mouse primers, respectively. Gene expression levels were calculated using the relative standard curve method, with normalisation to the geometric mean of the reference genes

A typical reaction contained 5μl of Master Mix, 0.6μl of both primers (10μM stock) or 0.2μl in the case of *Hmbs* primers and 4μl of 1/10 diluted cDNA to a total volume of 10μl.

Amplification was achieved in 40 cycles of the following conditions:

50oC for 2min, 95oC for 10min, 95oC for 15 sec 65oC for 45 sec, followed by the dissociation curve: 95 oC for 15 sec, 60 oC for 15 sec, 95 oC for 15 sec.

# PCR primer sequences

Human RT-PCR primers were as follows:

derived 1 CP1 chimeric transcript F (*DISC1* exon 6/7): aaggagcctccaggaaagaa derived 1 CP1 chimeric transcript R (*DISC1FP1* exon 3a): caagaaatgccaaagtgagtt derived 1 chimeric transcripts pan F (*DISC1* exon 6/7): aaggagcctccaggaaagaa derived 1 chimeric transcripts pan R (*DISC1FP1* exon 4): aaggagcctccaggaaagaa derived 11 chimeric transcripts F (*DISC1FP1* exon 2): gggacctggaattgaagaga derived 11 chimeric transcripts R (*DISC1* exon 9): gtctcctggtgctccacttc

*DISC2* F: ccctgaaggtgttgaacaagc

*DISC2* R: ctggaccctctgttgctgta *DISC1FP1* F: agagcaagaagagtggatgtgga *DISC1FP1* R: ccttgaggagtacgtcttaagctct

Human RT-QPCR primers were as follows:

*DISC1* F: ccagccttgcttgaagccaaaa *DISC1* R: tgaggagtccctccagcccttc *GAPDH* F: gagtccactggcgtcttcac *GAPDH* R: atgacgaacatgggggcatc *ACTB* F: gttacaggaagtcccttgccatcc

*ACTB* R: cacctcccctgtgtggacttggg *ERBB4* F: agagcccaccaattactcca *ERBB4* R: gtgtaacggtcccactagtca *PDE4B* F: cacggcgatgacttgattgt *PDE4B* R: tgtgggttgactctggagac *NRG1* F: aacaaagcatcactggctga *NRG1* R: aagacacatatgctccttcagttg

DRD2 F: aagggcacgtagaaggagac

DRD2 R: ggtcaccgtcatgatctcca

GRM5 F: ggagcttgattgtgatgcca

GRM5 R: tgcttctgtgagggcatga

CNTN5 F: tcaggcggtgctggaaata

CNTN5 R: ggctcccactgtctaactga

Mouse RT-PCR primers were as follows:

derived 1 chimeric transcripts pan F (*Disc1* exon 8): gtgctcaggtgagaagctgtg derived 1 chimeric transcripts pan R (*DISC1FP1* exon 4): gacccacagatggaatcgaa

Mouse RT-QPCR primers were as follows:

*Disc1* F: cctgccttgctggaagcca

*Disc1* R: cccttcccgctctgacgaca

Derived 1 chimeric transcripts pan F (*Disc1* exon 8): cctgccttgctggaagcca

Derived 1 chimeric transcripts pan R (*DISC1FP1* exon 4): aagacccacagatggaatcgaactg

*Cyclophilin* F: ggagatggcacaggaggaaag *Cyclophilin* R: gcccgtagtgcttcagcttgaa *Hmbs* F: ccctgaaggatgtgcctaccata *Hmbs* R: aaggtttccagggtctttccaa

Mouse genotyping was carried out using a multiplex reaction with the following primers:

*Disc1* F (intergenic sequence beyond final exon): cctgcatccacagacgtgc

*Disc1* R (intergenic sequence beyond final exon): cagtagtaagaaaagagacaaccccc Targeting vector F: ataacggtcctaaggtagcgagc

# PCR product sequencing

Where single PCR products were obtained, completed PCR reactions were treated with ExoSAP-IT (GE healthcare) prior to direct sequencing. Alternatively, where multiple products were generated (Supplementary Figure 3c), PCR products were excised from agarose gels and purified using the QIAquick Gel Extraction Kit (Qiagen). BigDye Terminator sequencing of PCR products was carried out using 1 μl BDv3.1, 1.5 μl 5X sequencing buffer, 1 μl DNA template and 6 μl dH2O. Reactions were cycled as follows; 96°C-1 min (1 cycle); 96°C-10 sec, 50ºC-5 sec, 60ºC-4 min (25 cycles); 4ºC hold. Sequencing chromatograms were analysed using Chromas (version 1.45).

# RNA sequencing

High density neuronal cultures (approximately 2 million cells per well in 12 well plates) were differentiated for 5 weeks. Immunofluorescence staining of parallel cultures was used to confirm correct NPC morphology and Nestin expression at the time of plating, and successful neuronal differentiation by assessing morphology and acquisition of βIII-tubulin expression at the time of harvesting, for every culture used. Three independent neuronal differentiations were performed per NPC line. Neurons were harvested in RNAlater (ThermoFisher Scientific), stored at -80oC, then processed in batches to extract the RNA. Each batch consisted of one triplicate per line to minimise batch effects.

All subsequent steps were performed by the Wellcome Trust Edinburgh Clinical Research Facility (www.wtcrf.ed.ac.uk). Total RNA samples were assessed on the Agilent Bioanalyser (Agilent Technologies, G2939AA) with the RNA 6000 Nano Kit (5067-1511) for

quality and integrity of total RNA, and then quantified using the Qubit 2.0 Fluorometer (Thermo Fisher Scientific Inc, Q32866) and the Qubit RNA BR assay kit (Q10210). Samples were also assessed for DNA contamination using the Qubit DNA HS assay Kit (catalogue Q32851).

Libraries were prepared from each total-RNA sample using the TruSeq Stranded Total RNA with Ribo-Zero Gold kit (RS-122-2301) according to the provided protocol.

500ng of total-RNA was processed to deplete rRNA before being purified, fragmented and primed with random hexamers. Primed RNA fragments were reverse transcribed into first strand cDNA using reverse transcriptase and random primers. RNA templates were removed and a replacement strand synthesised incorporating dUTP in place of dTTP to generate double-stranded cDNA. AMPure XP beads (Beckman Coulter, A63881) were then used to separate the double-stranded cDNA from the second strand reaction mix, providing blunt-ended cDNA. A single 'A' nucleotide was added to the 3' ends of the blunt fragments to prevent them from ligating to another during the subsequent adapter ligation reaction, and a corresponding single 'T' nucleotide on the 3' end of the adapter provided a complementary overhang for ligating the adapter to the fragment. Multiple indexing adapters were then ligated to the ends of the double-stranded cDNA to prepare them for hybridisation onto a flow cell, before 15 cycles of PCR were used to selectively enrich those DNA fragments that had adapter molecules on both ends and amplify the amount of DNA in the library suitable for sequencing.

Libraries were quantified by PCR using the Kapa Universal Illumina Library Quantification kit complete kit (KK4824) and assessed for quality using the Agilent Bioanalyser with the DNA HS Kit (5067-4626). Libraries were combined in three equimolar pools and sequencing was performed using the NextSeq 500/550 High-Output v2 (150 cycle) Kit (FC-404-2002) on the NextSeq 550 platform (Illumina Inc. SY-415-1002).

Sequences were aligned to the human reference genome Hg19 using the RNA-Seq Alignment v1.0 application (Illumina Inc.).

# RNA sequencing data analysis

Differential gene expression was analysed using DESeq2 from the R statistical package10. Differential exon expression was analysed using DEXSeq11. The three replicate samples used per cell line were obtained from independent neuronal differentiations which can follow slightly different trajectories. These replicates were therefore treated as biological rather than technical replicates. Due to sex imbalance in the samples all differentially expressed genes from the X and Y chromosomes were removed from the DESeq2 and DEXSeq analysis. For all downstream analyses, the full list of expressed genes was used as the background gene set, while an expression BaseMean cut-off of ten was applied to all differentially expressed data (corrected p<0.05) to minimise quantitation errors from genes expressed at very low levels. DESeq2 and DEXSeq data were combined and GO analysis was carried out using Gorilla ([http://cbl-gorilla.cs.technion.ac.il/).](http://cbl-gorilla.cs.technion.ac.il/)) Enrichment of GO terms categorised under Process, Function and Component was investigated. Ingenuity Pathway Analysis was carried out to complement the gene ontology analysis. Heat maps of gene expression were generated using R version 3.4.2 and RStudio version 1.0.143. Raw count data for all samples were together subjected to a regularised logarithm transformation10 using the DESeq2 package version 1.16.1. For each heat map, the transformed counts for each gene were normalised to Z-scores across all samples and subsequently visualised using the pheatmap package version 1.0.8 (cran.r-project.org/package=pheatmap).

# Peptide arrays

Peptide libraries were produced by automatic spot synthesis as described previously12, 13. Interaction of peptide spots with purified proteins was determined by overlaying the cellulose membranes with 10μg/ml recombinant protein. Membranes were incubated with protein that had been partially purified from 500μl of reticulocyte lysate and bound protein was detected by immunoblotting using specific primary antisera and a complementary HRP-coupled

secondary antibody. For alanine scanning the peptide was systematically mutated to alanine at every position, except existing alanine residues which were mutated to aspartate.

***In vitro* transcription and translation**

FLAG-DISC1 and FLAG-GluN1 C0-C1-C2 proteins were synthesized using the T3/T7 transcription/translation-coupled reticulocyte lysate system (Promega, Hampshire, UK). The translation product was partially purified by ammonium sulphate precipitation before use in peptide array analyses.

# Cell and tissue lysis

Cultured cells were lysed in ice-cold PBS containing 1% Triton X-100/10mM sodium fluoride/1mM DTT/2mM PMSF/5mM pyrophosphate/10% glycerol containing protease inhibitor cocktail (Roche) and phosphatase inhibitor cocktail II (Calbiochem). Lysates were solubilised by incubation for 30-60 minutes at 4oC on a rotary wheel and centrifuged at 13,000 rpm for 30min. Brain lysates were prepared from dissected tissue and homogenised in ice-cold PBS containing 1% Triton X-100/1% NP40/0.5% sodium deoxycholate/10% glycerol/1 mM DTT/ 10 mM β-glycerophosphate/10 mM NaF/phosphatase inhibitor cocktail II and 1V (Calbiochem) and 2X protease inhibitor cocktail (Roche), solubilised by incubation on a rotary wheel for 30 mins at 4oC and centrifuged at 45,000 rpm for 30 mins at 4oC to obtain the soluble fraction.

# Immunoblotting

Protein samples were separated by SDS-PAGE using NuPAGE polyacrylamide gels (Invitrogen), transferred onto PVDF membranes (GE Healthcare) using Trans-Blot SD Semi- Dry Transfer Cell (Bio-Rad) and blocked in 1% skimmed milk in T-TBS [50 mM Tris–HCl (pH 7.5), 150 mM NaCl and 0.1% Tween-20] at room temperature (RT). Incubation with primary antibodies was performed overnight at 4 °C. Blots were incubated with horseradish

peroxidase-conjugated secondary antibodies [Rabbit anti-mouse IgG HRP, Swine anti-rabbit IgG HRP or Rabbit anti-goat IgG HRP (DAKO)] for 45 min at RT and then protein bands were visualized by ECL or ECL-2 Western blotting substrate (Thermo Scientific). Some blots were stripped with Restore PLUS Western Blot Stripping Buffer (Thermo Scientific) and re- probed with appropriate antibodies, including secondary antibodies conjugated to alkaline phosphatase, with protein bands subsequently visualized using Western Blue (Promega).

Relative intensities of protein bands were quantified with ImageJ densitometry analysis.

# Subcellular Fractionation of Mouse Brain

Crude synaptosomes and light membrane fractions prepared as described previously14 have already been reported15. Briefly, C57BL/6 adult mouse brains were homogenized in a sucrose buffer solution [1mM HEPES (pH7.5), 0.32M sucrose, 1mM NaHCO3, 1mM MgCl2 Complete Mini Protease Inhibitor Tablet (Roche), Phosphatase Inhibitor Cocktail Set II (Calbiochem)] using a dounce tissue grinder. The S1 supernatant was obtained by centrifugation of the homogenates at 1,000*g* for 10 min and further fractionated into the S2 supernatant and the P2 crude synaptosomal pellets by centrifugation at 13,800 *g* for 10 min. To obtain the light membrane P3 pellets, the S2 supernatant was centrifuged at 100,000 *g* for 1 hr (Beckman TLA 100.3). The identity of the synaptosome fraction was confirmed previously by probing for PSD9515.

# Immunoprecipitation

Cultured cells were lysed in IP buffer [1% Triton X-100, with/without 0.1% SDS, 50 mM Tris– HCl (pH 7.5), 150 mM NaCl, Complete Mini Protease Inhibitor Tablet (Roche), Phosphatase Inhibitor Cocktail Set II (Calbiochem)]. Insoluble materials were removed by centrifugation at 100,000g. Crude synaptosomes/light membrane pellets were lysed in IP buffer and insoluble materials were removed by ultracentrifugation at 100,000g (Beckman TLA 100.3) for 30 min at 4°C. Cell lysates were pre-cleared with protein G-Sepharose beads (Sigma) alone for 1hr

at 4°C, and then incubated with appropriate antibodies overnight at 4°C with rotation. Protein G-Sepharose beads were added to the lysates and further incubated for 2 hrs. Immune complexes on the beads were washed with IP buffer three times and with 50mM Tris–HCl (pH 7.5) once. Bound proteins were eluted with SDS loading buffer and analyzed by immunoblotting.

# Antibodies

A new antibody specific for human DISC1 was generated as described previously15. Briefly, the C-terminus of human DISC1 isoform Lv (aa 669-832) was bacterially expressed as a GST-fusion protein and purified using Glutathione Sepharose 4B (GE Healthcare). New Zealand White rabbits were then immunized with the GST-human DISC1 669-832 protein and immune sera were collected (Eurogentec). Anti-DISC1 polyclonal antibodies were purified using Activated CH Sepharose 4B (GE Healthcare) to which the same antigen was covalently coupled. Antibodies that reacted with GST were removed from the immune sera using GST-coupled Activated CH Sepharose 4B prior to the affinity purification of anti-DISC1 antibodies. Generation and characterisation of an in-house antibody to mouse Disc1 has previously been described15, 16.

The following antibodies were used in the surface NMDAR expression study: GluN1 Mouse monoclonal IgG2a (BD Biosciences, 556308), GluN2A Rabbit polyclonal (Alomone Labs, AGC-002), GluN2B Rabbit polyclonal (Alomone Labs, AGC-003), βIII-Tubulin Rabbit polyclonal (Abcam, Ab18207), βIII-Tubulin/Tuj1 Mouse monoclonal IgG2a (Cambridge Bioscience, 801201) and PSD95 Mouse monoclonal IgG1k (ABR, MA1-046). Fluorescently labelled secondary antibodies were all AlexaFluor (Life Technologies): Goat anti mouse IgG1 488, Goat anti mouse IgG2a 647 and 594, Chicken anti rabbit 647, Goat anti rabbit 594.

The following antibodies were also used in this study: mouse monoclonal anti-FLAG M2 (Sigma F3165), anti-rabbit NR1 (GluN1, Sigma 8913), goat anti-HA (Abcam ab9134),

rabbit anti-Calreticulin (Sigma 06-661), mouse monoclonal anti-Vinculin (Abcam ab18058) and mouse monoclonal anti-Nestin (Millipore 10C2).

# Cell culture and transfection

All cultures were maintained at 37 °C and 5% CO2 in a humidified incubator. COS7 cells were grown in DMEM/10% foetal calf serum and passaged using a 1:1 trypsin:versene solution (Invitrogen). COS7 cells were transfected at 60-70% confluency using Lipofectamine 2000 (Life Technologies) according to the manufacturer’s instructions.

Hippocampal neurons were isolated from embryonic (E18) C57BL/6 mouse brains as described previously9, and grown in the presence of an astrocyte feeder layer. Astrocytes were purified from E18 C57BL/6 mouse brains as described previously17 and grown to confluence on rat tail collagen coated 30mm PTFE cell culture inserts (Millipore) in DMEM with GlutaMAX (Life Technologies) supplemented with 10% FCS. The astrocyte-containing cell culture inserts were then transferred to Poly-D-Lysine coated 6 well high precision (1.5H) glass bottom plates (In Vitro Scientific), and the medium was replaced with Neurobasal medium without phenol red supplemented with B27 and GlutaMAX (Life Technologies). Two days later, hippocampal neurons were seeded at low density (1x105/well) underneath each astrocyte-containing cell culture insert and in the presence of the astrocyte-preconditioned medium. When cultures were grown for longer than one week, the entire volume of medium (2 ml) in the astrocyte-containing insert was refreshed one week after plating, and every subsequent week afterwards. The genotype of each litter used to isolate hippocampal neurons was confirmed by PCR using the tail tips of the individual embryos.

For live imaging of GluN1-Dendra2 trafficking in dendrites, neurons were transfected at DIV7 with Lipofectamine 2000 (Life Technologies). A total of 1.75μg of endotoxin-free plasmid DNA (750 ng of pGluN1-Dendra2 plus 1μg of pCB6-HA-GluN2B) and 2μl of Lipofectamine 2000 were used per well. Plasmid DNA and Lipofectamine2000 were diluted in 50μl each of Neurobasal medium without phenol red, mixed thoroughly and incubated at 37 °C for 1 hour. 400μl of medium were removed from the astrocyte-containing insert

located in the well to be transfected, and used to dilute the transfection mix. The astrocyte- containing insert was then temporarily removed from the well, the entire volume of medium in the well was set aside and replaced with the diluted transfection mix, and then the astrocyte insert was returned to the well. After incubating for 4 hours at 37 °C and 5% CO2, the transfection mix was removed, and the pre-existing Neurobasal medium was returned to the well. The astrocyte insert was left in place, and replenished with fresh Neurobasal medium without phenol red supplemented with B27 and GlutaMAX. The transfected neurons were imaged on the day following transfection.

# Dendra2 NMDAR trafficking assay

Monomeric fluorescent protein Dendra2 exhibits green fluorescence (excitation/emission peaks: 490 nm/507nm) in its native state, and can be stably photoconverted to red fluorescence (excitation/emission peaks: 553nm/ 573nm) by exposure to intense 405nm light. Because GluN2 subunit availability is a limiting factor for receptor assembly18, GluN1- Dendra2-expressing neurons were co-transfected with an HA-GluN2B expression construct to maximise NMDAR assembly, and therefore trafficking outwith the ER.

C57/BL/6 hippocampal neurons expressing GluN1-Dendra2 plus HA-GluN2B were imaged at DIV8 (one day after transfection) using a Nikon C1 confocal microscope equipped with a Plan Apo 60x oil immersion objective (NA 1.4), and an environmental chamber.

Neurons were imaged in their original culture medium, and in the presence of the astrocyte- containing insert. Temperature and CO2 saturation were kept constant at 37°C and 5%, respectively, throughout the imaging period. To locate transfected neurons, the sample was observed with a FITC filter, using the minimum light intensity that still allowed green fluorescent signal to be obtained. This was necessary to prevent unwanted bulk Dendra2 photoconversion. After switching to confocal mode, the zoom factor and focus were rapidly adjusted while exciting the sample with a 488nm laser at low power (1%). To verify that no unwanted photoconversion had occurred, an image of the same field of view was then

obtained in the red channel by exciting the sample with a 561nm laser, confirming that no signal above background was detectable.

Transfected primary dendrites expressing GluN1-Dendra2 were selected and a small rectangular region of interest (ROI) was positioned in a central location along its longitudinal axis (Supplementary Figure 5c). This ROI was then used to target the 405nm laser to induce GluN1-Dendra2 photoconversion. The exact position and size of this ROI (termed the photoconversion ROI) was adjusted for each dendrite based on its size, thickness and brightness, with the aim of obtaining a sufficient amount of photoconverted protein to allow successful tracing. The average length along the dendrite of the photoconversion ROI was

~14µm, and did not vary significantly between experimental groups, ensuring comparability between measurements.

GluN1-Dendra2 photoconversion was induced by rapidly scanning a suitably positioned dendritic ROI with a 405nm laser (laser power: 10%, loops per second: 3, total stimulation time: 1 second, Supplementary Figure 5d). Immediately after photoconversion, acquisition of a time series was started manually (resulting in an effective delay of 10 seconds between photoconversion and acquisition of the first image frame). The time series consisted of 13 frames acquired at 15 second intervals, for a total imaging time of 180 seconds. This frame rate and total acquisition time were determined empirically, and were found to be the best compromise between fluorescence preservation over time (a higher frame rate would have induced more rapid photobleaching) and sufficiently accurate documentation of protein movement. The first frame contained both red (photoconverted GluN1-Dendra2) and green (native GluN1-Dendra2) channels, and all subsequent frames contained the red channel only, allowing movement of photoconverted GluN1-Dendra2 to be traced along the dendrite. Acquisition settings were as follows, and were kept consistent throughout the experiment. Frame size: 512 x 512 pixels, scan speed: 1 frame per second, pinhole size: 150μm (fully open), binning: 1x1, focus stabilisation: ON. To ensure comparability between images, laser power and detector settings were kept consistent throughout the experiment.

To quantify the movement of photoconverted GluN1 signal along the dendrite, a bespoke analysis algorithm was generated using iVision software (BioVision Technologies, script available on request). This algorithm uses the green (non-photoconverted) image of GluN1-Dendra2 to define the structure of the dendrite to be analysed (Supplementary Figure 5e-j). The resulting ‘skeleton’ corresponding to the longitudinal axis of the primary dendrite and all its branches is superimposed onto the red image, providing a guide or “track” along which the algorithm will then quantify the intensity of the red pixels. Such quantification is achieved by means of a circular analysis ROI whose centre moves along the track by incremental steps corresponding to one radius at a time. Since the data are deleted from the image after each measurement is taken, and before the analysis circle proceeds to the next location, the equivalent of half the area of this circle is analysed at each step, and no pixels are measured twice. The centre of the analysis circle is initially positioned in a location corresponding to the pixel coordinates of the geometric centre of the photoconconversion ROI. After measuring the intensity of the red pixels located within the central dendrite segment (or central bin), the first analysis circle moves along the track, starting from the proximal direction (towards the neuronal soma) and continuing until all proximal dendrite segments (bins) have been analysed through the time-lapse series. Next, the analysis circle repositions itself on the centre of the photoconversion ROI and then moves from here in the distal direction (away from the neuronal soma), proceeding along the distal tracks until all remaining dendritic segments have been analysed through the time-lapse series. Finally, two separate results tables are assembled for the proximal and distal sections of the dendrite.

The dendritic segment analysed at each step by the analysis circle is defined by its distance from the centre of the photoconversion ROI, its area, and the sum of the intensities of all red pixels measured within it throughout the time-lapse series. To correct for the effect of variations in dendrite size, total fluorescence intensity was divided by the area of the same segment. For each measured dendritic segment the width of each analysed dendritic segment corresponds to the radius of the analysis circle, which was arbitrarily set to 5 µm.

The 5μm bin therefore represents 5-10μm from the photoconversion ROI, and so on. Since

red fluorescence intensity did not change significantly over time in bins located beyond 40μm from the centre of the photoconversion ROI (data not shown), only bins located up to 40μm from the centre of the photoconversion ROI were analysed further.

A total of 37, 35 and 40 neurons were imaged per DISC1 expression construct (empty vector, FLAG-DISC1 or FLAG-DISC1-37W, respectively), each from three independent cultures. A total of 69 (*Disc1wt/wt*) and 68 (*Disc1Der1/Der1*) neurons were imaged

per genotype from three independent cultures. Imaging and analysis was carried out blind to

genotype.

Although several neurons were imaged per genotype, the number of neurons that produced usable data decreased in the furthest bins due to a combination of factors. First, not all neurons analysed had dendrites extending at least 40μm on both sides of the photoactivation centre. Second, some statistical outliers were excluded. Third, for each neuron, bins in which there was no clear relationship between fluorescence intensity and time were excluded from the analysis. This was determined by individually plotting their values. Three main classes of fluorescence intensity plot were obtained, corresponding to neurons in which 1) the fluorescence intensity peak was reached within the 10s preceding imaging onset (Supplementary Figure 5k), 2) the peak was reached within the bin (Supplementary Figure 5l), and 3) fluorescence intensity increased throughout the imaging period, most likely due to the peak moving too slowly to be captured within three minutes (Supplementary Figure 5m).

**Code availability**

The bespoke GluN1-Dendra2 motility analysis algorithm generated using iVision software

(BioVision Technologies) is available on request.

# NMDAR velocity estimates

Average velocities were estimated for the slow and fast NMDAR populations, taking into account the approximately 10s delay between photoconversion and imaging onset.

Fluorescence peak average velocities were determined from individual neuron fluorescence intensity plots as follows: The time to reach peak fluorescence was identified for each neuron per condition from the 10μm and 15μm bins. In neurons where fluorescence peaked in the 10s prior to imaging onset (Supplementary Figure 5k) peak fluorescence was set at 10s. All of these values were averaged per bin for each condition. Velocity estimates per bin per condition were then determined by dividing distance between the centre of the photoconversion ROI and the centre of the bin (for example, for the 10μm bin distance was 12.5μm), by time. The 10μm and 15μm bins were used for this analysis because the time to peak fluorescence could be determined for the majority of neurons in these bins, whereas in the 20μm bin, fluorescence did not peak in some neurons, thus any velocity estimates from this bin would be overestimated.

Maximum velocity estimates for the fast NMDAR population were determined from individual neuron fluorescence intensity plots as follows: Neurons from the 25μm, 30μm, 35μm and 40μm bins were examined. In these bins the majority of neurons exhibited a sigmoidal pattern of fluorescence intensity (Supplementary Figure 5n) interpreted as showing that fluorescence appeared in the bin after imaging onset. For these neurons a sigmoidal curve was fitted to the fluorescence intensity plot and the time at which fluorescence intensity first appeared on the linear part of the curve was taken as the time at which fluorescence reached the bin. In these bins a minority of neurons exhibited a pattern of steadily increasing fluorescence (Supplementary Figure 5m). In these neurons it was assumed that fluorescence had already reached the bin by imaging onset, thus fluorescence appearance in these neurons was set at 10s. All of these values were averaged per bin for each condition. Velocity estimates per bin per condition were then determined by dividing distance between the centre of the photoconversion ROI and the centre of the bin in question, by time.

# Immunocytochemistry

COS7 cells were fixed in 4% Paraformaldehyde (PFA) for 10 minutes and permeabilised with 0.2% Triton X-100 in PBS for 10 minutes. Blocking was carried out for 30 minutes in 3% BSA in PBS, followed by incubation with primary, then secondary, antibodies diluted in 3% BSA/PBS for 1 hour. All steps were carried out at room temperature.

To stain surface-expressed NMDAR on cultured hippocampal neurons, antibodies that selectively bind to the extracellular loop of the receptor subunits (either GluN1 Mouse monoclonal IgG2a, GluN2A Rabbit polyclonal or GluN2B Rabbit polyclonal) were directly added to the culture medium at a dilution of 1:100, and the living neurons were incubated at 37 °C for 10 minutes in a humidified incubator with 5% CO2. Neurons were then washed once with cold (4 °C) PBS, and fixed with room temperature 4% PFA for 10 minutes.

Neurons were incubated for 1 hour at room temperature with secondary antibodies to NMDAR subunit primary antibodies (Anti mouse IgG2a 594 or Anti rabbit 594), and then permeabilised with 0.1% Triton X-100 in PBS for 5 minutes. After blocking for 30 minutes with room temperature 3% BSA in PBS, neurons were incubated with primary antibodies to βIII-Tubulin or Tuj1 (Rabbit polyclonal or Mouse monoclonal, both 1:1000) and PSD95 (1:500) for 2 hours at room temperature. Finally, secondary antibodies to βIII-Tubulin and PSD95 (Anti rabbit 647 or Anti mouse 647 IgG2a and Anti mouse IgG1 488, respectively) were added for 1 hour at room temperature. After staining, the glass bottoms of the multi- well plates in which the neurons were grown were excised using a diamond tip pen and mounted on glass slides using Fluoroshield mounting medium (Sigma-Aldrich).

# Structured Illumination Microscopy image acquisition and analysis

Neurons were imaged from three independent cultures per genotype. Imaging and analysis was carried out blind to genotype. 3D Structured Illumination Microscopy (SIM) image stacks were acquired using a Nikon N-SIM super resolution microscope equipped with an Apo TIRF 100x oil immersion objective (NA 1.49) and an Andor DU-897 EMCCD camera. The camera settings were as follows, and were kept consistent throughout the experiment. Readout speed: 1 MHz, bit depth: 16 bit, EM gain multiplier: 300, conversion gain: 1x, binning: 1x1.

Laser power and/or exposure times were adjusted for each acquisition to produce the best signal to noise ratio, but keeping the grey levels in each image below 15,000. Each 3D-SIM stack consisted of 3 channels (green for PSD95, red for NMDAR subunits and far-red for βIII-Tubulin) and 17 slices, with the z-step size fixed at 120nm, for a total stack thickness of 2.04μm. To acquire each stack, the best focal plane was identified for each dendrite segment to be imaged, and 8 additional slices were acquired both above and below this

central focal plane. Dendrites with clearly distinguishable morphological features (main shaft, primary branches and secondary branches), and no spatial overlap with other dendrites from neighbouring neurons, were selected for image acquisition. For each individual neuron analysed, a single dendrite segment measuring between 32 and 46μm in length and located immediately after the first dendrite branching point was imaged. The image reconstruction parameters (Illumination Modulation Contrast (IMC), High Resolution Noise Suppression (HRNS) and Out of Focus Blur Suppression (OFBS) were empirically selected for each channel to generate the best quality images, with minimal artefacts and the best Fourier transforms. The following reconstruction parameters were applied, and kept consistent throughout the experiment. Green channel (PSD95): IMC 0.3, HRNS 0.1, OFBS 0.05; Red channel (NMDAR subunit): IMC 0.5, HRNS 0.1, OFBS 0.05; Far-red channel (βIII-Tubulin): IMC 1, HRNS 5, OFBS 0.05. Lateral image resolution was determined by measuring the full- width half maximum (FWHM) of the smallest punctate structures detectable in a single plane of the reconstructed image, and was equal to 120 nm in the green channel (PSD95) and 180 nm in the red channel (NMDAR subunits).

Images were first analysed automatically using the Surfaces function of Imaris 8.1 and 8.4 (Bitplane). 3D segmentation parameters were first tested extensively for each channel on a sample of images acquired over different imaging sessions, and visually validated by superimposing the resulting 3D reconstruction with the raw images to confirm that all structures visible in the latter were faithfully represented in the former. The optimised parameters were then applied to the analysis of the entire dataset. The measured parameters included the volume of the imaged dendrite segment (estimated from the 3D

rendering of βIII-Tubulin staining), as well as the number and volume of each individual punctum, and total volume of surface GluN1, GluN2A, GluN2B and of PSD95 proteins, based on the 3D reconstruction of their respective staining. Imaris 8.1 was also used to visualise and measure 3D co-localisation between each NMDAR subunit and PSD95 puncta. For intensity-based 3D co-localisation coefficients (Pearson’s and Mander’s), the above mentioned segmentation parameters were applied to each image to select the signal corresponding to either one of the NMDAR subunits and PSD95 puncta, and the intensity of the excluded voxels was considered below threshold and therefore set to zero.

# Statistical analysis

GraphPad Prism software was used for statistical analyses. Pairwise comparisons were assessed using t-tests. Mutant neuron trafficking data were analysed using two-tailed t-tests, paired for each timepoint, to examine whether fluorescence intensities differed overall between genotypes within each bin. DISC1 overexpression trafficking data were analysed using the Friedman repeated measures test with post-hoc Dunn’s testing. Otherwise, multiple comparisons were carried out using one-way ANOVA with post-hoc pairwise Bonferroni tests or, if data were not normally distributed, by Kruskal-Wallis with post-hoc pairwise Dunn’s tests, as stated for each figure. Interaction between genotype and the number of PSD95 nanodomains per cluster was tested using two-way ANOVA with post-hoc Bonferroni testing. The hypergeometric probability test was used to examine gene enrichment in the RNASeq dataset. Statistical outliers, defined as being more than three standard deviations from the mean, were removed in one round from the GluN1-Dendra2 trafficking data and the surface expression data. All values are presented as mean ± SEM.


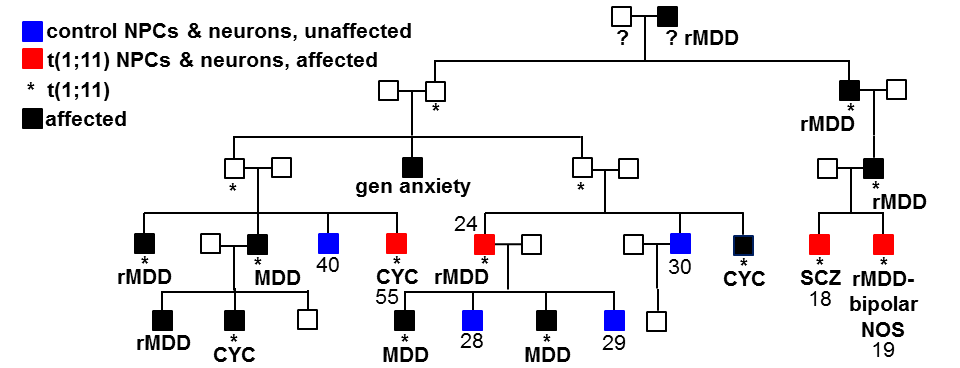


**Supplementary Figure 1.** Translocation family pedigree indicating members from whom neural precursors and neurons were studied. rMDD, recurrent major depressive disorder; MDD, major depressive disorder; SCZ, schizophrenia; bipolar-NOS, bipolar disorder not otherwise specified; CYC, cyclothymia; ?, translocation carrier status unknown


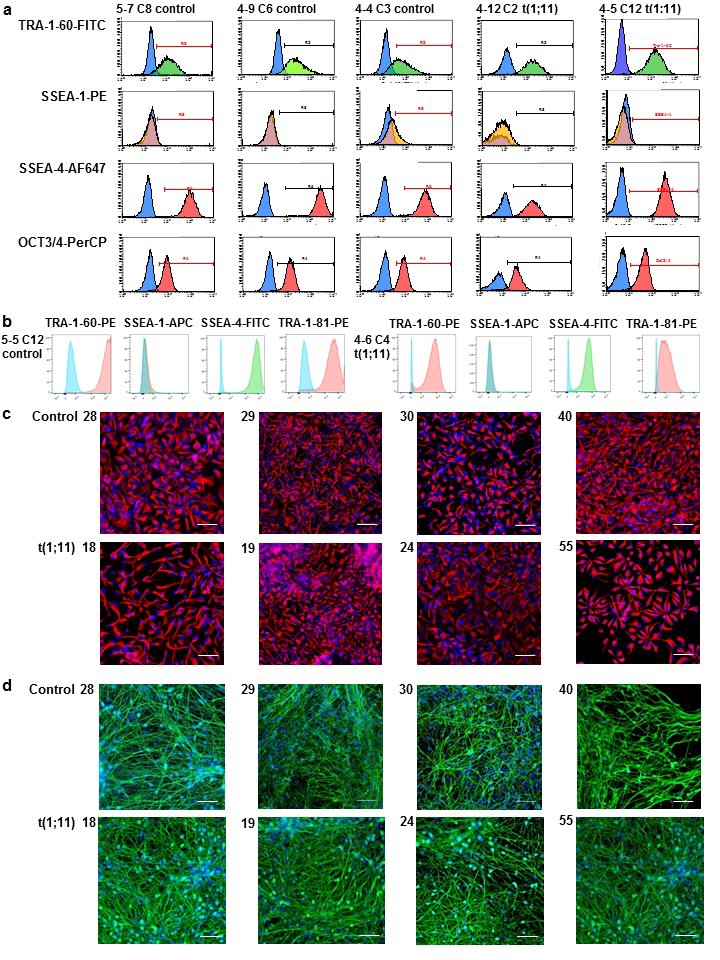


**Supplementary Figure 2.** IPSC and neural cell controls. (**a**)(**b**) IPSC lines were subjected to flow cytometry using fluorescently labelled antibodies specific for cell surface markers. Flow cytometry detected the pluripotent stem cell markers TRA-1-60, SSEA-4 and OCT3/4, or

TRA-1-60, SSEA-4 and TRA-1-81, respectively, but not the differentiation marker SSEA-1. y- axes, cell counts; x-axes, fluorescence; blue peaks, immunoglobulin isotype controls. (**c**) Representative examples of NPC lines stained for the neural stem/progenitor cell marker NES (red), with nuclei visualised using DAPI (blue). (**d**) Representative examples of neurons generated by differentiating NPC lines for five weeks, stained for the neuronal marker βIII- tubulin (green), with nuclei visualised using DAPI (blue). Scale bars, 5μm


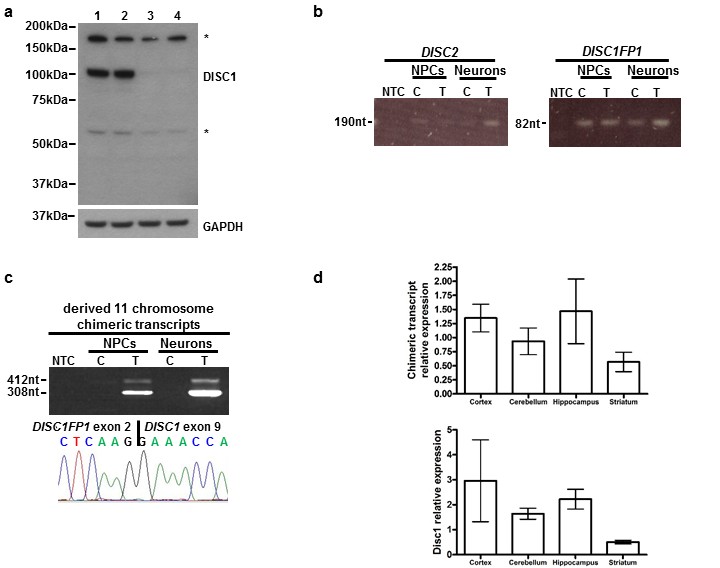


**Supplementary Figure 3.** An antibody to detect human DISC1, and additional data from human NPCs & neurons, and from the *Der1* mouse. (**a**) Protein lysates from HEK293 cells transfected with previously tested human DISC1-specific siRNA duplexes 9, were immunoblotted and probed to demonstrate the specificity of a new antibody for full-length DISC1, or with GAPDH antibody. Only one species, at approximately 100kDa, is reduced by siRNA treatment. 1, mock transfection (no siRNA); 2, control siRNA; 3, DISC1-siRNA #2; 4, DISC1-siRNA #5; * non-specific bands. (**b**) Detection of *DISC2* and *DISC1FP1* expression by RT-PCR in neural precursors (NPCs) and neurons. Amplification specificity was confirmed by PCR product sequencing (data not shown). Expression of both genes was too low to be accurately quantified in these cell types. (**c**) Detection of chimeric transcripts from the derived 11 chromosome by RT-PCR followed by sequencing of the major product. Note that there are two products due to alternative splicing 6. (**d**) Quantification of chimeric (upper) or wild-type *Disc1* (lower) transcript expression in *Disc1Der1/Der1* or *Disc1wt/wt* adult mouse

brain regions, respectively. N=4 per brain region per genotype; NTC, non-template control; C, control with normal karyotype; T, translocation carrier; error bars represent SEM


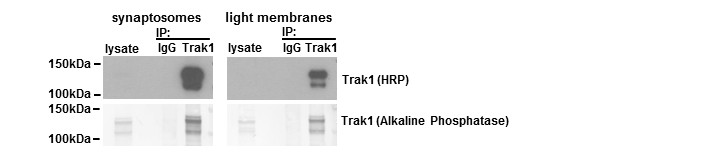


**Supplementary Figure 4.** Trak1/GluN2B co-immunoprecipitation. Trak1 was immunoprecipitated from adult mouse brain synaptosome and light membrane fractions as shown in **Figure 3k**. Trak1 was detected in the lysates using secondary antibody conjugated to alkaline phosphatase.


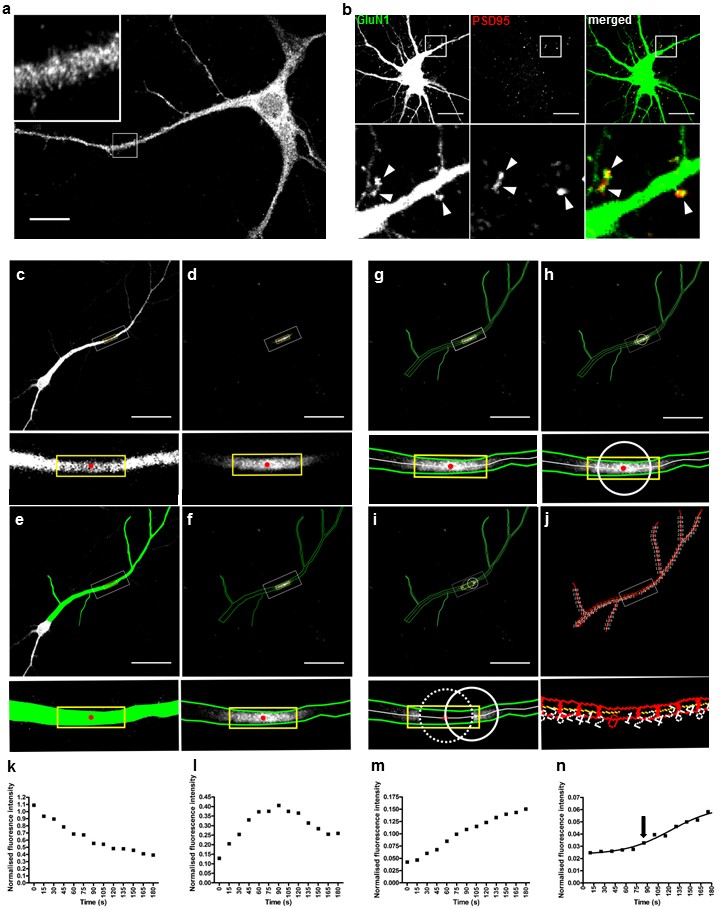


**Supplementary Figure 5.** NMDAR trafficking assay controls and analysis of photoconverted GluN1-Dendra2 movement along a dendrite. (**a**) Endogenous GluN1 detected in wild-type DIV8 hippocampal neurons by immunofluorescence exhibits a fine granular appearance. scale bar, 15μm (**b**) GluN1-Dendra2 (native green Dendra2 fluorescence) co-localises with

endogenous PSD95 in DIV14 hippocampal neurons within dendritic spines. scale bar, 20μm, arrowheads point to synapses (**c**)(**d**) Green fluorescent (non photoconverted) and red fluorescent (photoconverted) GluN1-Dendra2 in the first image frame captured after photoconversion. scale bars in c-j, 50μm (**e**) The dendrite area to be analysed is defined by a segmentation mask (green) applied on the green channel. (**f**) The area delimited by the segmentation mask is shown as a green line in the corresponding red channel image. Only the intensity of the red pixels located within the segmentation mask is measured. (**g**) A ‘skeleton’ (white line) corresponding to the longitudinal axis of the primary dendrite and all its branches is superimposed on the red image, providing a guide along which the algorithm will quantify the intensity of the red pixels. (**h**) At the start of the analysis, the analysis circle (white circle, 10µm in diameter) is placed on the geometric centre of the photoconversion ROI. Red pixels delimited by the intersection of the measuring circle with the dendrite outline (green) are measured through the time series. (**i**) The analysis circle moves one step along the dendrite in the distal direction. The new position of the centre of the analysis circle is defined by the intersection point between its previous position (dotted circle) and the dendrite skeleton (white line). The previous position of the circle (dotted circle) has been measured, and the corresponding data have been deleted. (**j**) Once the measuring circle has covered the entire dendritic area included in the image, an image showing all generated segments and their distance (in pixels) from the geometric centre of the photoconversion ROI is displayed. Mean intensity measured in all subsequent segments is normalised to the mean intensity measured in this first segment. (**k**) Example fluorescence intensity plot from a 5μm bin where fluorescence peaked within the 10 seconds prior to imaging onset. (**l**) 15μm bin where fluorescence peaked within the imaging period. (**m**) 25μm bin where fluorescence increased steadily because the peak was not reached during the imaging period. (**n**) 25μm bin where fluorescence appearance above background was delayed for 90s. This plot illustrates the use of sigmoidal curves to identify when fluorescence appears, as indicated by the arrow.

Yellow rectangles represent the photoconversion ROI (14µm wide on average); red dots indicate the geometric centre of the photoconversion ROI; white rectangles indicate enlarged areas


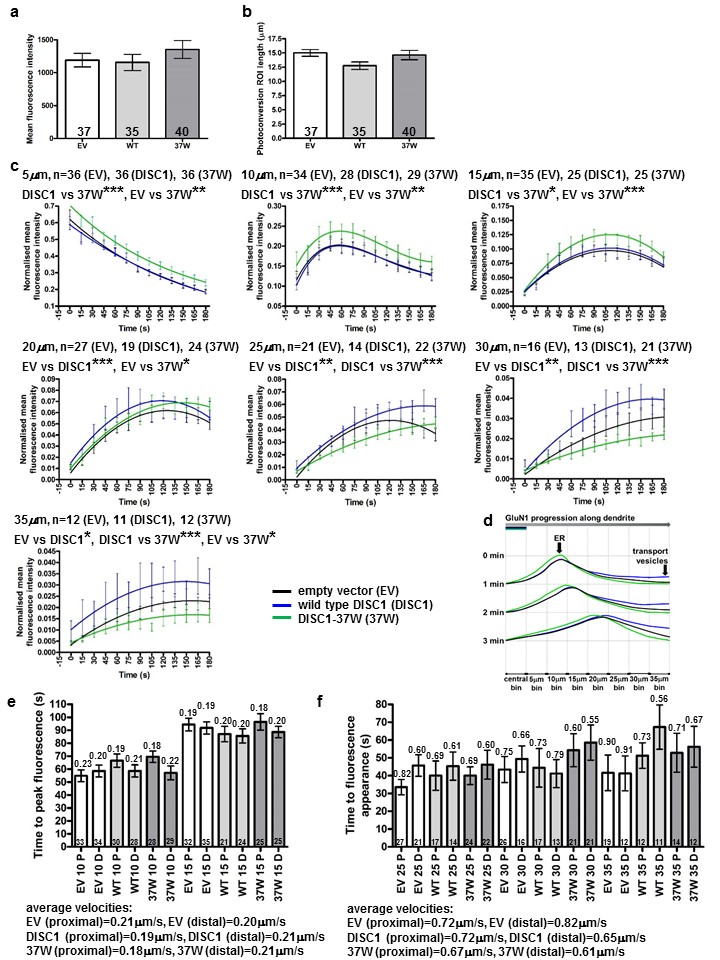


**Figure 6.** Altered distal dendritic NMDAR trafficking in mouse hippocampal neurons overexpressing DISC1 or DISC1-37W. (**a**)(**b**) Mean red fluorescence intensity, a, or ROI

dendritic length, b, in the central bin in *Disc1wt/wt* and *Disc1Der1/Der1* DIV8 neurons was equal at time zero following photoconversion. (**c**) Quantification of fluorescence intensity over time in successive 5μm dendritic bins distal to the centre of the photoconversion ROI. Data analysed by Friedman repeated measures test (p<0.0001, p<0.0001, p<0.0001, p=0.0006, p<0.0001, p<0.0001, p<0.0001 for the 5μm to 35μm bins, respectively) with Dunn’s post-hoc

test. (**d**) Model of dendritic GluN1-Dendra2 motility. Photoconverted GluN1-Dendra2 progresses in a wave-like fashion, with the fastest and slowest moving GluN1-Dendra2 at the leading and trailing edges, respectively, and the bulk travelling as the ‘crest’. (**e**) Fluorescence peak velocity estimates for the 10μm and 15μm bins. Average time to peak fluorescence was converted to velocity, indicated above each bar. Average velocities were

determined from the two bins. (**f**) Fast-moving GluN1-Dendra2 maximum velocity estimates for the 25μm-40μm bins. Average time to fluorescence appearance was converted to velocity, indicated above each bar. Average velocities were determined from the four bins. EV, empty vector; DISC1, wild-type DISC1; 37W, DISC1-37W; error bars represent SEM; *** p<0.001; ** p<0.01; *p<0.05; n indicated on graphs


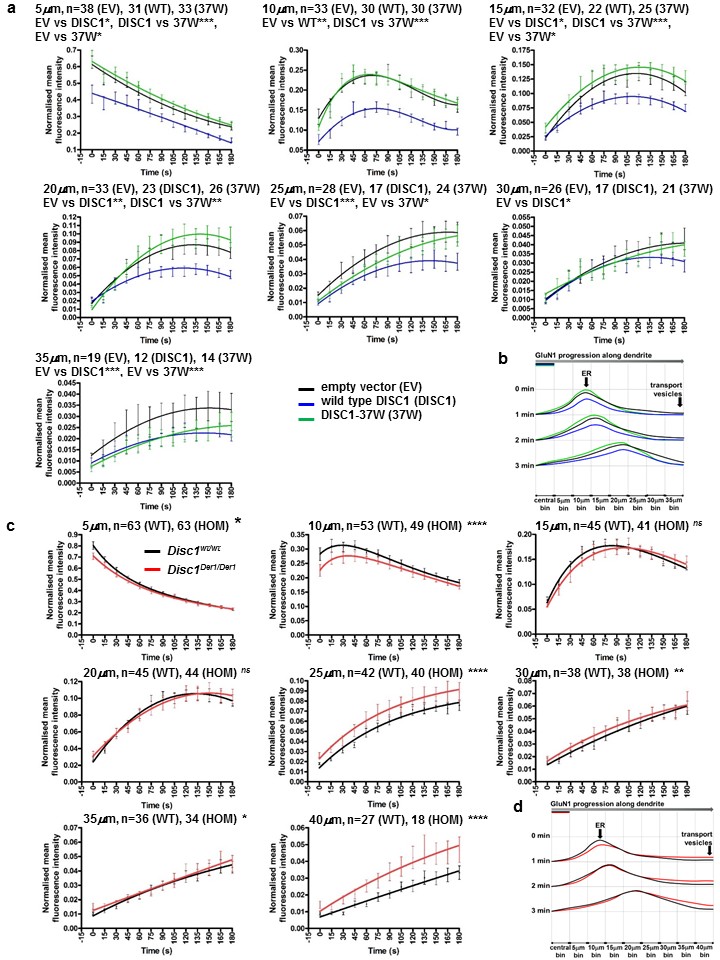


**Supplementary Figure 7.** Altered proximal dendritic GluN1-Dendra2 motility due to DISC1 overexpression or the *Der1* mutation. (**a**) Effect of DISC1 overexpression. Quantification of fluorescence intensity over time in successive 5μm dendritic bins proximal to the centre of the photoconversion ROI. Mean fluorescence intensity at each time point within each bin is normalised to mean fluorescence intensity in the central bin at time zero per neuron. Total neuron numbers from three independent cultures are indicated. To determine whether fluorescence intensity differed between the expression constructs within each bin, data were analysed by Friedman repeated measures test (p<0.0001, p<0.0001, p<0.0001, p=0.0008, p<0.0001, p=0.02, p<0.0001, for the 5μm to 35μm bins, respectively) with Dunn’s post-hoc testing. (**b**) Model of dendritic GluN1-Dendra2 motility as described in **Figure 4e**. Total neuron numbers from three independent cultures are indicated. EV, empty vector; DISC1, wild-type DISC1; 37W, DISC1-37W; error bars represent SEM; *** p<0.001; ** p<0.01;

*p<0.05 (**c**) Effect of the *Der1* mutation. Quantification of fluorescence intensity over time in successive 5μm bins proximal to the centre of the photoconversion ROI. Mean fluorescence intensity at each time point within each bin is normalised to mean fluorescence intensity in the central bin at time zero per neuron. Total neuron numbers from three independent cultures are indicated. To determine whether fluorescence intensity differed between the genotypes within each bin, data were analysed by paired two tailed t-test to make comparisons at each timepoint. (**d**) Model of dendritic GluN1-Dendra2 motility as described in **Figure 4e**. WT, *Disc1wt/wt*; HOM, *Disc1Der1/Der1*; error bars represent SEM, **** p<0.0001, ** p<0.01, * p<0.05, *ns* not significant


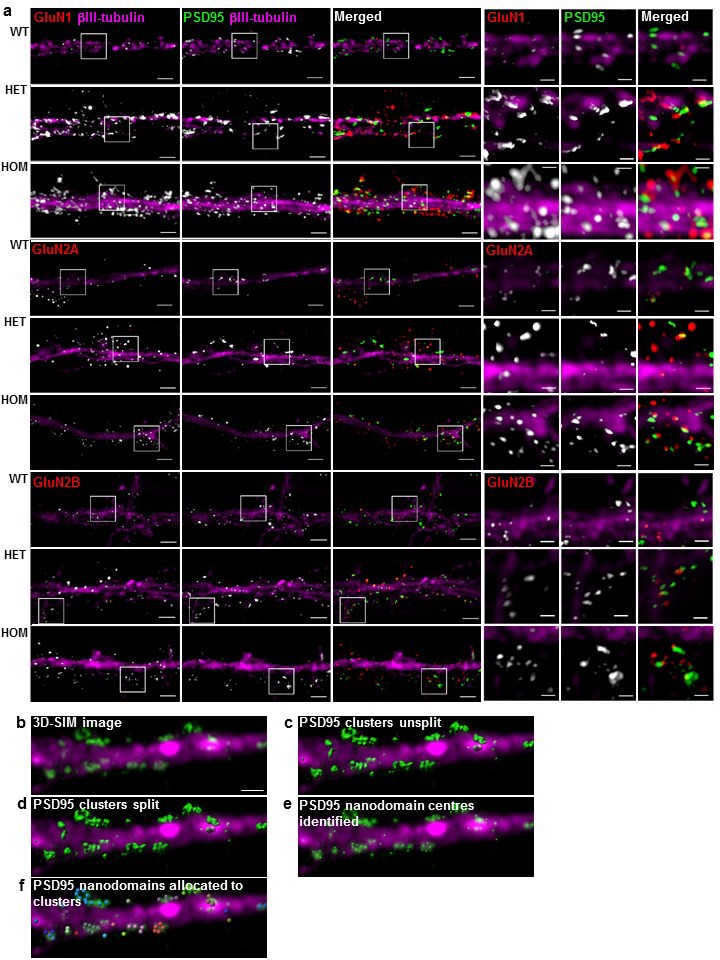


**Supplementary Figure 8.** Dendritic NMDAR and PSD95 expression in hippocampal neurons. (a) 3D-SIM images of surface GluN1, GluN2A or Glun2B, and total PSD95 and βIII-

tubulin (Tuj1). WT, *Disc1wt/wt*; HET, *Disc1(wt)/Der1*; HOM, *Disc1Der1/Der1*; scale bars, 2μm in main images, 0.6μm in enlarged insets indicated by white *boxes (b) 3D-SIM image of a dendrite segment showing PSD95 (green) and βIII-tubulin (Tuj1, magenta). Scale bar B-F, 1µm (c)*

Identification of Imaris surfaces for PSD95. These three-dimensional surfaces are counted

and their volume is quantified by the software. (d) PSD95 surfaces split into individual

nanodomains. (e) Identification of the centre of each individual nanodomain, and conversion

to Imaris spots using a bespoke MATLAB (MathWorks) XTension script. (f) Individual

nanodomains are assigned to clusters, and the number per cluster is counted using the

Imaris spots MATLAB XTension ‘Split into Surface Objects’.


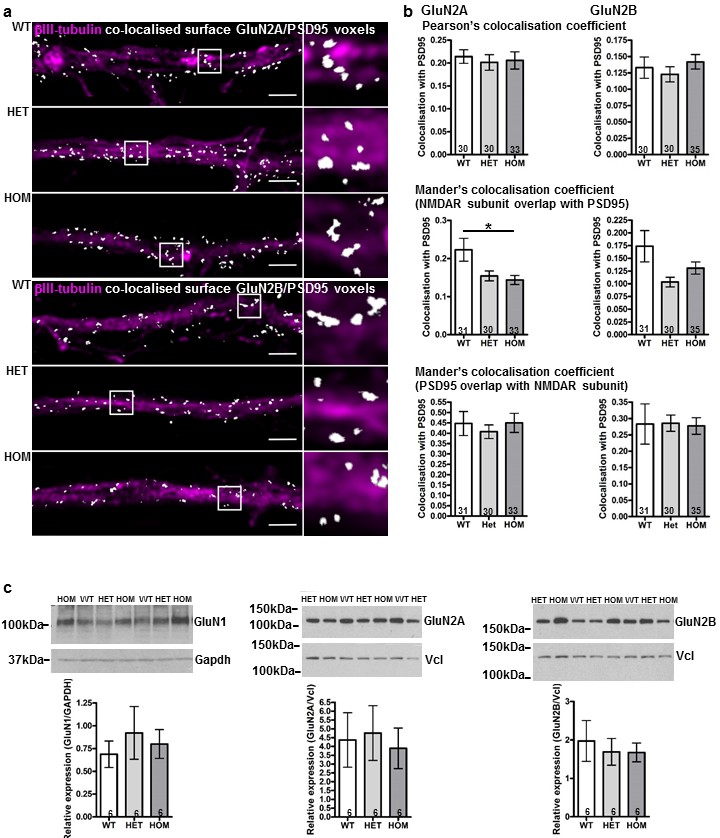


**Supplementary Figure 9.** NMDAR subunit GluN2A and GluN2B co-localisation with the post-synaptic density marker PSD95. (**a**) Reconstructed 3D-SIM images of dendrites. Co- localised voxels, which contain signal from both PSD95 and surface-expressed GluN1 are shown in white. White boxes indicate enlarged regions. Scale bars, 3μm (**b**) Pearson’s coefficient indicates overall correlation of each signal. Mander’s coefficients measure the

amount of subunit fluorescent signal co-localised with total PSD95 signal, and vice versa. Data were analysed by Kruskal-Wallis (p=0.02 for GluN2A Mander’s M1, p=0.06 for GluN2B Mander’s M1) followed by Dunn’s multiple comparison test. WT, *Disc1wt/wt*; HET, *Disc1(wt)/Der1*; HOM, *Disc1Der1/Der1*; error bars represent SEM; * p<0.05 (**c**) Immunoblots of hippocampus lysates were prepared from nine week mice, and probed with antibodies specific for GluN1. GluN2A and GluN2B NMDAR subunits, followed by loading controls Gapdh and Vcl. Subunit expression relative to the loading controls was quantified using densitometry. Data were analysed by Kruskal-Wallis test and no significant differences were found WT, *Disc1wt/wt*; HET, *Disc1(wt)/Der(1)*; HOM, *Disc1Der(1)/Der(1*); error bars represent SEM; n indicated on graphs


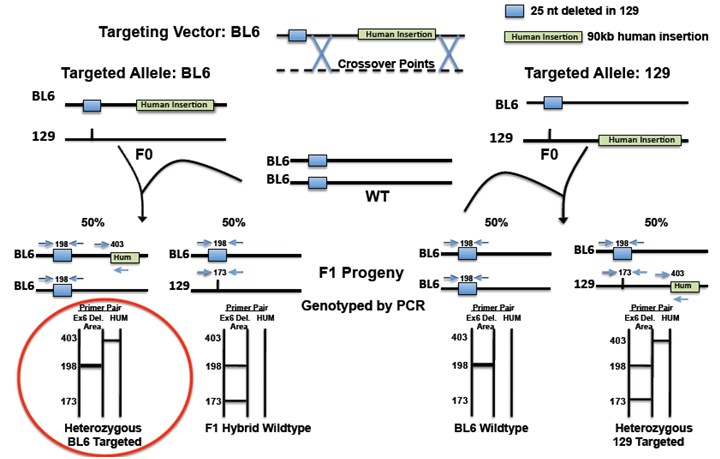


**Supplementary Figure 10.** Screening for C57BL/6 targeted clones. PCR flanking the area of the naturally occurring *Disc1* deletion (25 base pairs)5 in strain 129S6SvEv distinguishes allele targeting. F1 progenies derived from C57BL/6 targeted clones produce a PCR product of 198nt from exon 6 and a 403 nt product from the human insertion.

**Supplementary Table 2. Imaris image analysis data.** Numbers of NMDAR subunit and PSD95 puncta counted for each genotype plus corresponding dendritic volumes. WT, *Disc1wt/wt*; HET, *Disc1wt/Der1*; HOM, *Disc1Der1/Der1*

|  | **WT** | **HET** | **HOM** |
| --- | --- | --- | --- |
| **GluN1**  **dendritic volume (μm3)** | 5,032  3,773 | 7,579  2,740 | 12,477  4,259 |
| **GluN2A**  **dendritic volume (μm3)** | 6,428  4,584 | 6,158  3,687 | 7,618  3,628 |
| **GluN2B**  **dendritic volume (μm3)** | 6,522  4,523 | 6,769  2,805 | 10.959  5,336 |
| **PSD95 unsplit PSD95 split** | 14,669  23,306 | 14,025  22,775 | 20,953  33,170 |
| **total dendritic volume (μm3)** | 12,881 | 9,232 | 13,223 |

# SUPPLEMENTARY REFERENCES

1. Okita K, Matsumura Y, Sato Y, Okada A, Morizane A, Okamoto S et al. A more efficient method to generate integration-free human iPS cells. Nat Methods. 2011;8:409-412.
2. Chambers SM, Fasano CA, Papapetrou EP, Tomishima M, Sadelain M, Studer L. Highly efficient neural conversion of human ES and iPS cells by dual inhibition of SMAD signaling. Nat Biotechnol. 2009;27:275-280.
3. Bilican B, Livesey MR, Haghi G, Qiu J, Burr K, Siller R et al. Physiological normoxia and absence of EGF is required for the long-term propagation of anterior neural precursors from human pluripotent cells. PLoS One. 2014;9:e85932.
4. Dechiara TM, Poueymirou WT, Auerbach W, Frendewey D, Yancopoulos GD, Valenzuela DM. VelociMouse: fully ES cell-derived F0-generation mice obtained from the injection of ES cells into eight-cell-stage embryos. Methods Mol Biol. 2009;530:311-324.
5. Koike H, Arguello PA, Kvajo M, Karayiorgou M, Gogos JA. Disc1 is mutated in the 129S6/SvEv strain and modulates working memory in mice. Proc Natl Acad Sci USA. 2006;103:3693-3697.
6. Eykelenboom JE, Briggs GJ, Bradshaw NJ, Soares DC, Ogawa F, Christie S et al. A t(1;11) translocation linked to schizophrenia and affective disorders gives rise to aberrant chimeric DISC1 transcripts that encode structurally altered, deleterious mitochondrial proteins. Hum Mol Genet. 2012;21:3374-3386.
7. Jeyifous O, Waites CL, Specht CG, Fujisawa S, Schubert M, Lin EI et al. SAP97 and CASK mediate sorting of NMDA receptors through a previously unknown secretory pathway. Nature Neurosci. 2009;12:1011-1019.
8. Malavasi EL, Ogawa F, Porteous DJ, Millar JK. DISC1 variants 37W and 607F disrupt its nuclear targeting and regulatory role in ATF4-mediated transcription. Hum Mol Genet. 2012;21:2779-2792.
9. Ogawa F, Malavasi EL, Crummie DK, Eykelenboom JE, Soares DC, Mackie S et al.

DISC1 complexes with TRAK1 and Miro1 to modulate anterograde axonal mitochondrial trafficking. Hum Mol Genet. 2014;23:906-919.

1. Love MI, Huber W, Anders S. Moderated estimation of fold change and dispersion for RNA-seq data with DESeq2. Genome Biol. 2014;15:550.
2. Anders S, Reyes A, Huber W. Detecting differential usage of exons from RNA-seq data. Genome Res. 2012;22:2008-2017.
3. Baillie GS, Adams DR, Bhari N, Houslay TM, Vadrevu S, Meng D et al. Mapping binding sites for the PDE4D5 cAMP-specific phosphodiesterase to the N- and C- domains of beta-arrestin using spot-immobilized peptide arrays. Biochem J. 2007;404:71-80.
4. Bolger GB, Baillie GS, Li X, Lynch MJ, Herzyk P, Mohamed A et al. Scanning peptide array analyses identify overlapping binding sites for the signalling scaffold proteins, beta-arrestin and RACK1, in cAMP-specific phosphodiesterase PDE4D5. Biochem J. 2006;398:23-36.
5. Clapcote SJ, Lipina TV, Millar JK, Mackie S, Christie S, Ogawa F et al. Behavioral phenotypes of Disc1 missense mutations in mice. Neuron. 2007;54: 87-402.
6. Ogawa F, Murphy LC, Malavasi EL, O'Sullivan ST, Torrance HS, Porteous DJ et al.

NDE1 and GSK3beta Associate with TRAK1 and Regulate Axonal Mitochondrial Motility: Identification of Cyclic AMP as a Novel Modulator of Axonal Mitochondrial Trafficking. ACS ChemNeurosci. 2016;7:553-564.

1. Chandran JS, Kazanis I, Clapcote SJ, Ogawa F, Millar JK, Porteous DJ et al. Disc1 variation leads to specific alterations in adult neurogenesis. PLoS One. 2014;9:e108088.
2. Kaech S, Banker G. Culturing hippocampal neurons. Nature Prot. 2006;1:2406-2415.
3. Horak M, Petralia RS, Kaniakova M, Sans N. ER to synapse trafficking of NMDA receptors. Front Cell Neurosci. 2014;8:394.
